# Potential of photoacoustic imaging for assessing tumor perfusion in intraocular tumors and high-resolution choroidal microvascular imaging: from technical advances to clinical translation

**DOI:** 10.3389/fonc.2026.1901235

**Published:** 2026-08-24

**Authors:** Huarong Wu, Cancan Huang, Shu Xu, Yanming Tian

**Affiliations:** 1 Department of Ophthalmology, Anqing Municipal Hospital, Anqing, China; 2 Department of Ultrasound, China Rongtong Medical Health Group Anqing 116 Hospital, Anqing, China; 3 Department of Ophthalmology, Southwest Medical University Affiliated Tianfu Hospital, Meishan, China

**Keywords:** choroidal melanoma, intraocular tumors, optical coherence tomography angiography, oxygen saturation, photoacoustic imaging, retinoblastoma

## Abstract

Accurate diagnosis and treatment-response assessment for intraocular tumors, typified by choroidal melanoma and retinoblastoma, require characterization of tumor microvascular architecture and oxygenation. However, commonly used ophthalmic modalities—such as optical coherence tomography angiography (OCTA), contrast-enhanced ultrasound (CEUS), and magnetic resonance imaging (MRI)—are limited by penetration depth, reduced spatial resolution at depth, and/or insufficient functional specificity, which can hinder noninvasive evaluation of deep hypoxic regions associated with aggressiveness and treatment resistance. Photoacoustic imaging (PAI), which combines optical absorption contrast with ultrasound detection, may help address these gaps under eye-specific safety constraints. Based on a structured literature search and a narrative/scoping synthesis of ophthalmic evidence supported by cross-disciplinary methodological studies, this review summarizes advances and translational considerations of PAI for intraocular tumor assessment. Current evidence—predominantly preclinical and early ophthalmic reports—suggests that PAI may extend vascular visualization beyond the optical scattering limit and enable label-free depiction of intratumoral vascular features in thicker lesions. Multispectral PAI can also be used to estimate three-dimensional oxygenation maps, including apparent oxygen saturation (sO_2_), although quantification in melanin-rich tumors remains sensitive to optical fluence attenuation and spectral unmixing bias. We discuss multimodal strategies that combine PAI with OCTA, MRI, and positron emission tomography (PET), and highlight key barriers, including standardized sO_2_ validation, ocular motion artifacts, and stringent ocular laser safety limits. Overall, PAI is best positioned as a complementary tool rather than a replacement for established ophthalmic imaging, and its clinical value requires rigorous validation.

## 1 Introduction

Assessment of tumor perfusion and vascularity in intraocular tumors—particularly choroidal melanoma and retinoblastoma—is essential for diagnosis, prognostic stratification, and treatment-response monitoring. Tumor angiogenesis has been associated with malignant progression, invasive and metastatic potential, and response to conservative therapies. However, currently available ophthalmic imaging modalities remain limited in their ability to comprehensively characterize deep tumor microvascular architecture and the associated microenvironment.

Optical coherence tomography angiography (OCTA) provides high-resolution visualization of retinal and superficial choroidal microvasculature, but optical scattering limits its penetration depth (often ~1–3 mm), thereby constraining evaluation of intratumoral flow networks in thicker lesions (1). Contrast-enhanced ultrasound (CEUS) provides dynamic perfusion-related information but relies on exogenous microbubble agents and lacks molecular specificity (2). Magnetic resonance imaging (MRI), particularly dynamic contrast-enhanced magnetic resonance imaging (DCE-MRI), can depict macroscopic perfusion patterns, yet spatial resolution is often insufficient for detailed assessment of small intraocular lesions and typically requires gadolinium-based contrast agents, which carry potential risks in selected populations (3). Consequently, clinical decision-making may rely disproportionately on macroscopic anatomical changes or delayed hemodynamic proxies, which can be less sensitive to early microenvironmental adaptation. These constraints may hinder noninvasive assessment of deep tumor regions where perfusion changes may co-exist with hypoxia-related adaptation, complicating individualized treatment planning (e.g., anti-vascular endothelial growth factor (VEGF) therapy and radiotherapy) and early response evaluation.

Photoacoustic imaging (PAI) offers a hybrid approach that leverages the photoacoustic effect: nanosecond-pulsed laser excitation of endogenous chromophores induces thermoelastic expansion, generating acoustic waves detected by ultrasound transducers. This mechanism combines optical absorption contrast with ultrasound-enabled tissue penetration (4, 5). Recent advances, including multispectral optoacoustic tomography (MSOT) and photoacoustic microscopy (PAM), have further enabled the extraction of hemoglobin-related functional information, including oxygen saturation and other oxygenation-related metrics (6, 7). With exogenous contrast agents, PAI can also be extended to molecularly targeted imaging for applications such as image-guided interventions and drug-delivery evaluation (8).

For intraocular tumors, PAI is technically aligned with key histopathologic features of choroidal melanoma, which is often melanin-rich and highly vascularized, providing strong endogenous absorbers. Nevertheless, melanin-dominant absorption can intensify optical fluence attenuation and spectral interference, potentially biasing oxygenation-related quantification and reducing robustness in pigment-rich tumors under eye-specific safety constraints (8, 9). Although *in vivo* clinical evidence for intraocular tumors remains limited, cross-disciplinary studies support the methodological feasibility of PAI for vascular and functional imaging. For example, multispectral and multimodal PAI have been validated in small-animal studies of cerebral blood flow and vascular contrast agents (10) and have been explored to address contrast–resolution trade-offs relative to purely optical and purely ultrasound-based imaging (11, 12). PAI has also been investigated as a complementary tool in breast imaging (13) and has been widely used in oncologic research to interrogate tumor structure, function, and molecular composition, and to monitor treatment response (14, 15). However, these precedents primarily provide methodological support, and their transferability to intraocular tumors requires dedicated ophthalmology-specific validation, as the spherical ocular geometry, motion characteristics, and strict ocular laser safety constraints limit direct extrapolation from systemic tissues.

Several barriers must be addressed before routine ophthalmic implementation. These include unavoidable ocular motion artifacts that challenge acquisition stability (16), the inherent depth–resolution trade-off in deep choroidal imaging, and the need for standardized calibration and validation of reconstruction and quantification algorithms. Translational experience with interventional and multimodal photoacoustic imaging in deep tissues (17) and inflammatory disease settings (18, 19) may provide useful engineering and methodological reference points.

From an evidence standpoint, ophthalmic PAI literature remains dominated by preclinical and translational studies, and—to date—human evidence has been largely feasibility-oriented, with a stronger focus on non-tumor ocular targets (e.g., retinal/choroidal vasculature or anterior segment applications) rather than on large intraocular tumor cohorts (11). Reports directly involving intraocular masses are comparatively scarce and are largely confined to early proof-of-concept descriptions, small case series, and/or ex vivo analyses (20). Accordingly, the limited availability of direct human tumor data is a key reason why current assessments must rely on ophthalmology-specific translational studies (including pigmented large-animal models) together with carefully bounded extrapolation from the cross-disciplinary photoacoustic oncology literature (21).

In this context, this review synthesizes current evidence on PAI for choroidal vascular imaging and intraocular tumor perfusion assessment, evaluates multimodal integration strategies, and discusses key translational challenges relevant to clinical translation. The evidence selection framework utilized in this review is outlined in the subsequent section.

### 1.1 Search strategy

We conducted a structured literature search in PubMed (MEDLINE), Embase, and Web of Science Core Collection for articles published from 1 January 2014 to 15 July 2026; the searches were last updated on 15 July 2026 during the manuscript revision. Searches were performed in titles and abstracts (and author keywords where available). The search strategy used in this review is presented for the PubMed database in **Table 1**, and the corresponding search strategies for Embase and the Web of Science Core Collection are provided in **Tables 1**, **2**.

**Table 1:** PubMed database search strategy.

No.	Search query
#1	“Photoacoustics”[Mesh]
#2	“Eye Neoplasms”[Mesh]
#3	“Retinal Neoplasms”[Mesh]
#4	“Uveal Neoplasms”[Mesh]
#5	“Photoacoustic Imaging”[tiab] OR “Photoacoustic Tomography”[tiab] OR “Photoacoustic Microscopy”[tiab] OR “Photoacoustic Ophthalmoscopy”[tiab] OR “PA Imaging”[tiab] OR “PAI”[tiab] OR “PAT”[tiab] OR “Photoacoustic”[tiab] OR “Optoacoustic Imaging”[tiab] OR “Optoacoustic Tomography”[tiab] OR “Photo-acoustic”[tiab] OR “Photo acoustic”[tiab]
#6	“Intraocular Tumor*”[tiab] OR “Intra-ocular Tumor*”[tiab] OR “Eye Tumor*”[tiab] OR “Ocular Tumor*”[tiab] OR “Retinoblastoma”[tiab] OR “Uveal Melanoma”[tiab] OR “Choroidal Melanoma”[tiab] OR “Choroidal Hemangioma”[tiab] OR “Choroidal Metastasis”[tiab] OR “Intraocular Neoplasm*”[tiab] OR “Ocular Neoplasm*”[tiab]
#7	#1 and #5
#8	#2 or #3 or #4 or #6
#9	#7 and #8

Following deduplication, titles and abstracts were screened and potentially eligible articles underwent full-text evaluation. Studies were included if they involved clinical or highly relevant preclinical intraocular imaging and primarily assessed tumor perfusion, microvascular architecture, and/or oxygenation-related endpoints. Methodological papers addressing ophthalmology-relevant translational barriers (ocular laser safety, motion artifacts, and optical fluence correction) were also included. To reflect rapid advancements, we prioritized literature published within the last five years for synthesis, while seminal ophthalmic PAI studies published before 2014 were additionally identified through hand-searching and citation tracking and included when relevant. Conference abstracts, preprints, and unpublished data were excluded.

### 2.2 Limitations of current imaging for intraocular tumor perfusion assessment and the rationale for PAI

#### 2.2.1 OCTA: limited optical penetration and incomplete visualization of deep tumor vasculature

OCTA is widely used to assess retinal and superficial choroidal microvascular networks because it is noninvasive, offers high spatial resolution, and typically does not require exogenous contrast agents. However, OCTA relies on motion contrast (decorrelation signals derived from erythrocyte motion), and optical scattering limits its effective imaging depth (1). In choroidal melanoma, where tumor thickness often exceeds 2 mm, OCTA may predominantly depict superficial vascular features and provide limited characterization of deeper intratumoral microvascular architecture. This surface-weighted sampling can hinder evaluation of angiogenic heterogeneity and the spatial distribution of hypoxic cores implicated in tumor invasion, metastasis, and treatment resistance; moreover, OCTA primarily provides morphology- and flow-related information and does not directly provide quantitative oxygen saturation (sO_2_) without additional oximetry approaches, limiting its ability to distinguish adequately oxygenated perfusion from perfused yet hypoxic regions (21). During longitudinal monitoring after radiotherapy or anti-angiogenic therapy, OCTA may therefore capture relatively delayed morphological vascular changes rather than earlier functional alterations linked to microenvironmental adaptation. In addition, segmentation errors, shadowing, and projection artifacts can further complicate interpretation in elevated or highly pigmented lesions, and these issues may be amplified when fixation is unstable.

#### 2.2.2 CEUS: limited molecular specificity and restricted microvascular resolution

CEUS enhances vascular signals via intravenously administered microbubble agents and their nonlinear acoustic scattering, enabling dynamic evaluation of macroscopic perfusion. In intraocular tumor assessment, CEUS can provide perfusion patterns and kinetic readouts (e.g., time–intensity curve features). A key limitation is its lack of molecular specificity: because contrast depends on the intraluminal distribution of microbubbles, CEUS cannot resolve biochemical states in blood, including the oxygenation state of hemoglobin (i.e., it cannot distinguish oxyhemoglobin from deoxyhemoglobin), and thus cannot directly map intratumoral oxygenation gradients (2). In addition, although microbubble agents are generally considered safe, hypersensitivity reactions can occur, and in pediatric populations, such as those with retinoblastoma (RB), the use of exogenous contrast agents may warrant more stringent ethical and safety considerations (2). Therefore, despite its value for macroscopic perfusion assessment, CEUS provides limited information on oxygenation-related microenvironmental heterogeneity and has restricted resolution for fine microvascular architecture. Practical deployment can also be constrained by operator dependence and variability in acoustic windows, which may reduce quantitative comparability across visits.

#### 2.2.3 DCE-MRI: spatiotemporal resolution constraints and contrast-agent–related considerations

DCE-MRI quantifies signal changes after gadolinium-based contrast administration and can model perfusion and vascular permeability, yielding parameters such as the volume transfer constant (*K*_trans_). For small intraocular tumors, DCE-MRI faces several constraints. First, routine spatial resolution is typically on the millimeter scale, substantially larger than the micrometer-scale dimensions of the choriocapillaris and intratumoral microvessels (22). Second, gadolinium-based agents carry recognized risks in selected populations, and for pediatric patients requiring repeated follow-up, cumulative risk–benefit considerations warrant careful evaluation. Third, temporal resolution is relatively limited (often minutes per acquisition series), restricting real-time capture of transient hemodynamic changes after radiotherapy or pharmacologic interventions. Collectively, these spatiotemporal and contrast-agent–related considerations constrain the role of DCE-MRI for real-time, high-precision perfusion assessment in intraocular tumors. In practice, scan time, motion sensitivity, and workflow burden (including the need for patient cooperation or sedation in some settings) can further limit repeated use. For a concise cross-modality comparison of outputs and limitations, see **Table 2**. **Table 2** summarizes modality-specific outputs and key limitations for intraocular tumor perfusion/microenvironment assessment, highlighting the depth–information trade-offs and the lack of direct oxygenation mapping in routine workflows.

**Table 2:** Comparison of imaging modalities for intraocular tumor perfusion and microenvironment assessment.

Modality	Agent	Primary outputs	Depth/Res	O_2_ mapping	Key limitations	Ref
OCTA	No	Superficial MV & flow	~1–3 mm (scattering)	No	Deep MV limited; artifacts in pigmented lesions	(1)
CEUS	MB	Perfusion kinetics	Macro-scale	No	Lacks O_2_ specificity; IV MB risks; operator-dependent	(2)
DCE-MRI	Gd	Perfusion (K_trans_)	~mm/~min	Indirect	Low MV detail; Gd risks; workflow/motion burden	(22)
PET	^18^F-FDG	Global metabolism	Low spatial res	No	Poor micro-localization; radiation; requires fusion	(10)
PAI	Often No	MV + HbT + apparent sO_2_	mm→cm (MPE-limited)	Yes (apparent)	Pigment/spectral bias in sO_2_; motion; needs validation	(1, 20, 21)

Depth/Res entries are conceptual (as stated in the main text) rather than head-to-head benchmarks. For PAI, sO_2_ represents an apparent estimate and may be biased by fluence attenuation, pigment-related spectral mixing, and motion.

OCTA, optical coherence tomography angiography; CEUS, contrast-enhanced ultrasound; DCE-MRI, dynamic contrast-enhanced magnetic resonance imaging; PET, positron emission tomography; PAI, photoacoustic imaging; MV, microvasculature; MB, microbubbles; HbT, total hemoglobin (proxy); sO_2_, oxygen saturation; *K*_trans_, volume transfer constant; MPE, maximum permissible exposure; ^18^F-FDG, ^18^F-fluorodeoxyglucose; Gd, gadolinium-based contrast agent.

#### 2.2.4 PAI: potential for combined perfusion and oxygenation assessment

PAI is a hybrid modality in which pulsed laser excitation induces thermoelastic expansion and generates broadband acoustic signals that are detected by ultrasound transducers, thereby partly addressing the limited penetration of purely optical imaging and the low intrinsic contrast of conventional ultrasound. In general tissue settings, imaging depth can range from millimeters to centimeters depending on wavelength and optical properties; however, in ophthalmic applications, eye-specific laser safety constraints, including the maximum permissible exposure (MPE), and strong ocular/choroidal absorption are expected to impose stricter limits on deliverable fluence and effective depth. Even within these constraints, PAI may provide greater depth sensitivity than OCTA and may help probe deeper choroidal layers and thicker tumors, potentially enabling visualization of deep abnormal vascular networks. In practice, depth performance may vary substantially across patients and lesion phenotypes, and negative or equivocal findings should not be interpreted as absence of vascularity without considering fluence attenuation and system-dependent sensitivity.

PAI also enables functional imaging. With multispectral excitation, PAI can provide vascular morphology along with hemoglobin-contrast–based signals, supporting the estimation of local apparent sO_2_ and an integrated “structure–function” assessment (1). This capability may help distinguish adequately oxygenated perfusion from perfused yet hypoxic regions, although quantification remains sensitive to fluence-related bias and spectral unmixing uncertainty, particularly in pigment-rich lesions. Direct ophthalmic clinical evidence remains limited; therefore, current support is largely methodological and cross-disciplinary (20). For example, PAI has been explored for evaluating myocardial oxygenation/ischemia (23) and for resolving endogenous and exogenous chromophore information in pediatric contexts (2). Longitudinal monitoring of vascular morphology and sO_2_ dynamics has also been proposed as a means of capturing early functional changes during radiotherapy or systemic therapy, potentially informing treatment adaptation in the presence of oxygenation heterogeneity (21). A further limitation is that “functional” readouts are not necessarily specific to hypoxia and can be confounded by changes in blood volume, hematocrit, or inflammation-related hyperemia, complicating mechanistic interpretation.

#### 2.2.5 A shared gap: dissociation between structural and functional information

A shared limitation across intraocular tumor perfusion assessment is the dissociation between structural and functional information. OCTA is often surface-weighted in thicker lesions and provides limited access to deep tumor features such as vascular mimicry or severely hypoxic cores, which may reduce its standalone value for malignancy stratification and early response prediction. CEUS can show rapid wash-in and wash-out patterns suggestive of malignancy; however, because of limited molecular specificity, it cannot distinguish hypoxia-driven angiogenesis (e.g., mediated by hypoxia-inducible factor-1α (*HIF-1α*)) from perfusion increases secondary to benign inflammation (21). Similarly, when differentiating highly vascular choroidal melanoma from choroidal hemangioma with secondary changes (e.g., thrombosis or fibrosis), DCE-MRI perfusion parameters may overlap, limiting diagnostic specificity. In addition, across modalities, perfusion surrogates can be sensitive to acquisition settings and operator-dependent factors, reducing comparability across sites and longitudinal visits.

**Figure 1:**
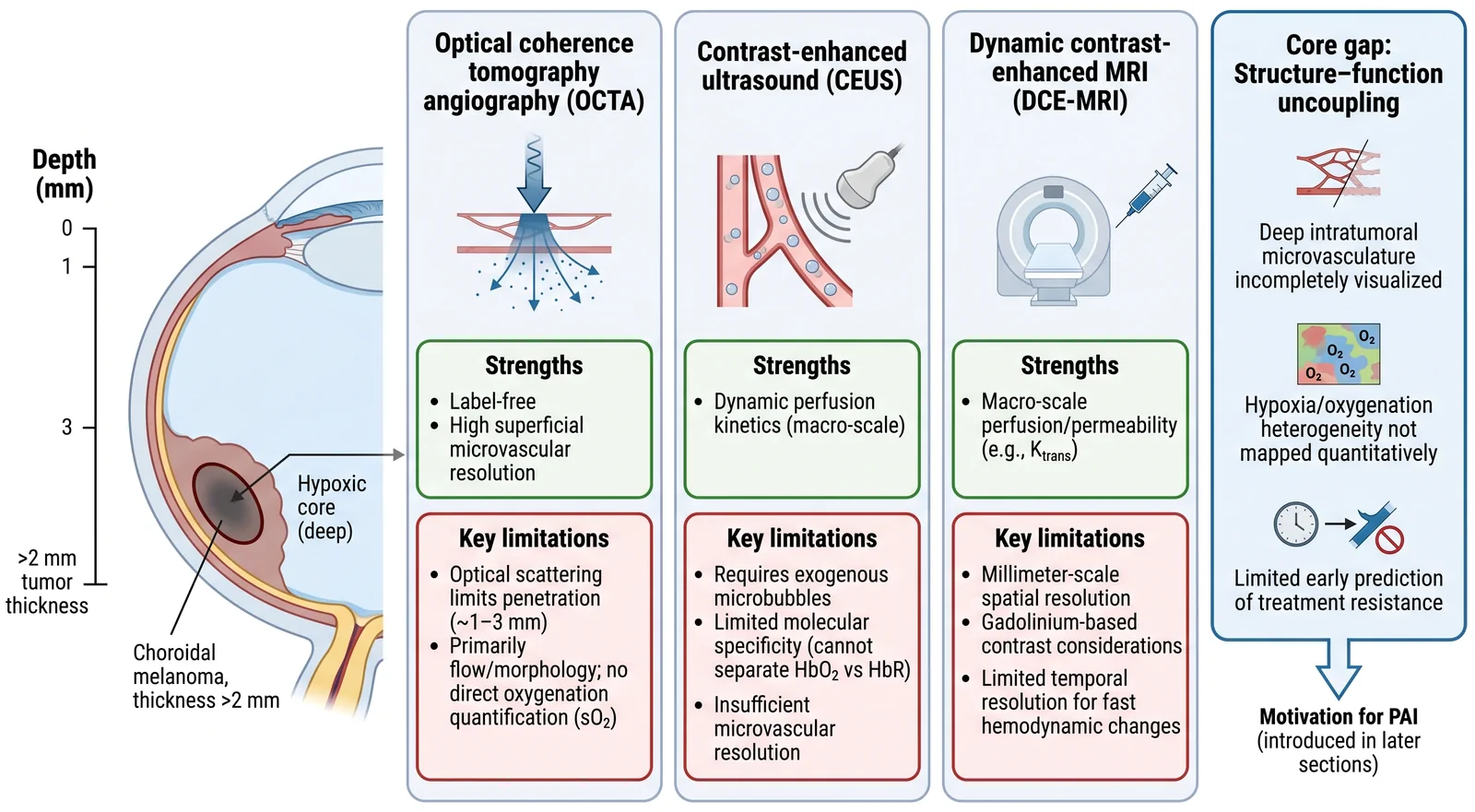
Limitations of current ophthalmic imaging and the “structure–function uncoupling” gap in intraocular tumor perfusion assessment. Schematic comparison of OCTA, CEUS, and DCE-MRI for assessing perfusion and the tumor microenvironment in intraocular tumors (e.g., choroidal melanoma). OCTA provides high-resolution superficial microvascular morphology/flow-related contrast but is limited in interrogating deeper intratumoral vasculature and hypoxic cores in thicker lesions due to optical scattering. CEUS depicts macro-perfusion kinetics but relies on exogenous microbubbles and lacks specificity for hemoglobin oxygenation. DCE-MRI provides macro-scale perfusion/permeability surrogates (e.g., *K*_trans_) but is constrained by spatial/temporal resolution and contrast-agent–related considerations. Collectively, these limitations lead to incomplete, noninvasive characterization of deep microvasculature and oxygenation heterogeneity, motivating PAI as a complementary approach. (Schematic generated with the assistance of Gemini 3.1.). OCTA, optical coherence tomography angiography; CEUS, contrast-enhanced ultrasound; DCE-MRI, dynamic contrast-enhanced magnetic resonance imaging; PAI, photoacoustic imaging; sO_2_, oxygen saturation; HbO_2_, oxyhemoglobin; HbR, deoxyhemoglobin; *K*_trans_, volume transfer constant.

Importantly, tumor invasion, metastasis, and treatment resistance are shaped not only by macroscopic blood flow but also by the mismatch between oxygen delivery and metabolic demand (i.e., a hypoxic microenvironment) (21). Because OCTA, CEUS, and DCE-MRI do not directly provide quantitative maps of hypoxia, hemoglobin-contrast–based functional imaging with PAI, particularly sO_2_-related estimation, may help address this information gap and provide complementary cues for characterizing intratumoral microenvironmental heterogeneity. However, this conceptual advantage will translate into clinical utility only if sO_2_ estimates are sufficiently repeatable and interpretable under ocular MPE constraints and if incremental value beyond existing workflows can be demonstrated in well-designed ophthalmic cohorts. As summarized in **Figure 1**, current modalities involve an intrinsic trade-off between superficial microvascular depiction, macro-perfusion kinetics, and depth-resolved oxygenation-related information, resulting in incomplete noninvasive assessment of deep intratumoral microvasculature and oxygenation heterogeneity in thicker lesions. This “structure–function uncoupling” gap provides a primary rationale for considering PAI as a complementary tool.

#### 2.2.6 Technical compatibility between PAI and intraocular tumor pathophysiology

From an anatomical and pathophysiological perspective, PAI is biophysically compatible with several key features of intraocular tumors. Choroidal tissue and melanoma lesions are enriched in melanin and hemoglobin—both strong absorbers in the near-infrared range—and can serve as endogenous photoacoustic contrast sources (24). This composition may enable delineation of tumor margins and visualization of vascular features and pigment distribution without exogenous tracers. At the same time, strong melanin-related absorption can increase optical fluence attenuation and background interference, reducing signal detectability at depth and biasing hemoglobin-based functional readouts in pigment-rich lesions (25). Moreover, a high pigment burden may compress the effective dynamic range, such that superficial signals dominate reconstructions, while deeper microvascular signals become less reliable.

Intraocular tumors also exhibit marked microenvironmental heterogeneity, with hypervascular regions intermingled with severely hypoxic areas. Multispectral PAI may enable three-dimensional oxygenation mapping via apparent sO_2_ estimates by leveraging the differential absorption spectra of oxyhemoglobin and deoxyhemoglobin, which could be relevant to radioresistance-related evaluation (21). In addition, the optically transparent ocular media (cornea, aqueous humor, lens, and vitreous) provide an optical pathway for delivering excitation light to the posterior segment with relatively low scattering and absorption. However, this advantage is coupled with stringent safety constraints because the cornea and retina have low damage thresholds to laser exposure, imposing strict requirements on system design and validation. As highlighted in strategic roadmaps for clinical translation (9), broader prospective studies are needed to define safe exposure parameters and validate diagnostic performance and long-term safety before PAI can move from mechanistic microcirculation research to routine ophthalmic clinical use (1). Notably, even when safety is ensured, practical implementation will require robust motion handling and standardized calibration to avoid site-dependent variability that could otherwise undermine clinical confidence.

### 2.3 Key strengths of PAI for assessing tumor perfusion and the microenvironment in intraocular tumors

**Figure 2:**
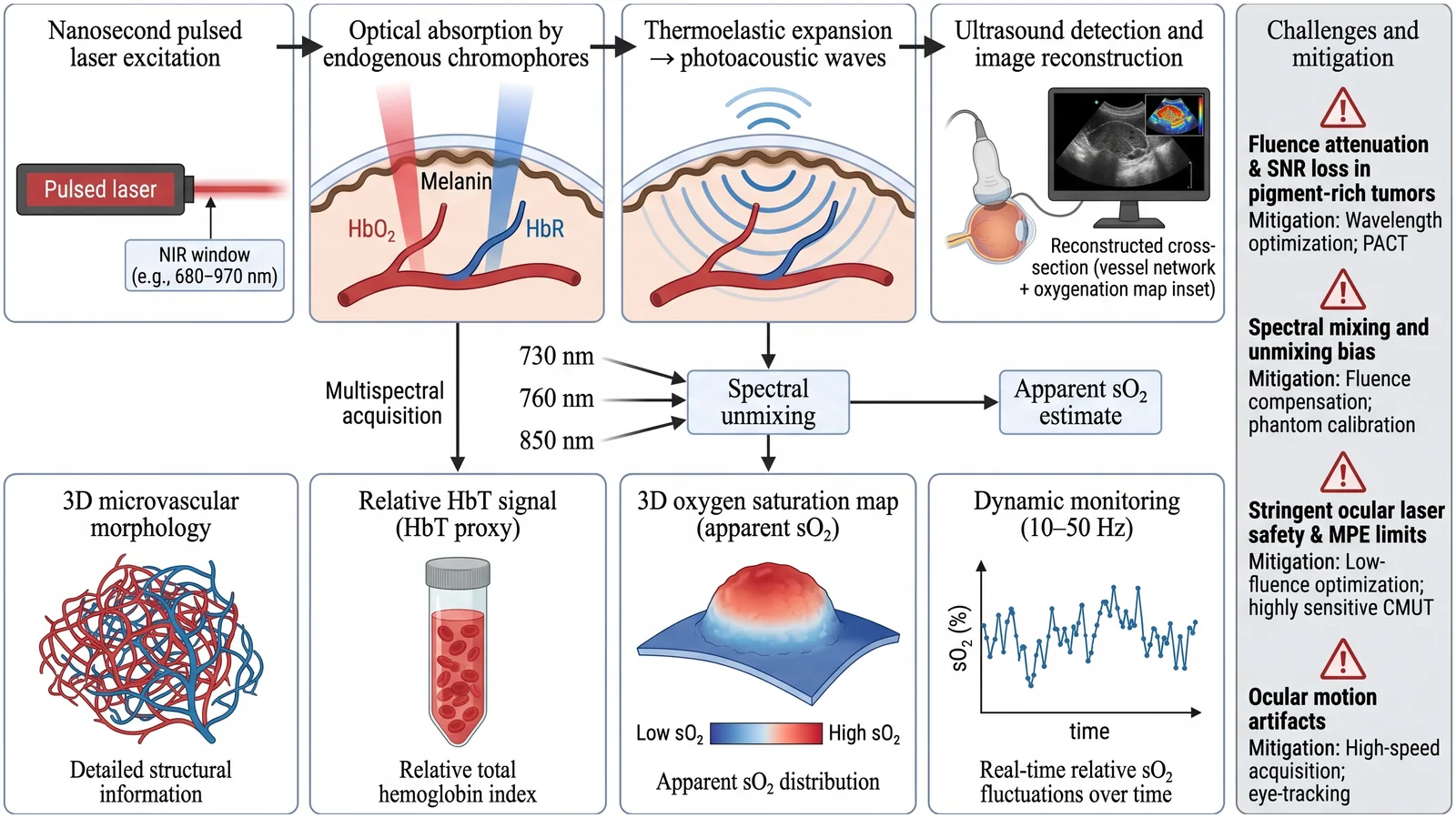
Photoacoustic imaging workflow, quantitative outputs, and major sources of uncertainty under ophthalmic constraints. Schematic overview of the PAI pipeline from nanosecond-pulsed laser excitation to absorption by endogenous chromophores (e.g., HbO_2_, HbR, and melanin), thermoelastic expansion generating photoacoustic waves, ultrasound detection, and image reconstruction. Multispectral acquisition and spectral unmixing enable (i) 3D microvascular morphology, (ii) relative total hemoglobin-related signals (HbT proxy), and (iii) apparent oxygen saturation (apparent sO_2_) maps, with potential for dynamic monitoring at moderate frame rates. Major ophthalmology-relevant challenges are summarized on the right, including fluence attenuation–driven SNR loss in pigment-rich tumors, spectral mixing/unmixing bias, stringent ocular laser safety and MPE limits, and ocular motion artifacts, together with representative mitigation strategies (wavelength optimization, PACT configurations, fluence compensation, phantom-based calibration, low-fluence optimization, optimized detector arrays such as CMUT, and eye-tracking–assisted motion handling). (Schematic generated with the assistance of Gemini 3.1.). NIR, near-infrared; HbO_2_, oxyhemoglobin; HbR, deoxyhemoglobin; HbT, total hemoglobin (proxy); sO_2_, oxygen saturation; SNR, signal-to-noise ratio; PACT, photoacoustic computed tomography; MPE, maximum permissible exposure; CMUT, capacitive micromachined ultrasound transducer.

This section summarizes the core capabilities of PAI for intraocular tumor assessment, focusing on depth-resolved microvascular depiction, oxygenation-related readouts derived from spectroscopic acquisition, and major sources of uncertainty under ocular constraints (e.g., fluence attenuation and motion). These elements form the basis for the subsequent discussion on performance trade-offs and translational requirements. An overview of the PAI workflow and representative quantitative outputs is provided in **Figure 2**. Under ophthalmic constraints, sO_2_ should be interpreted as an apparent estimate whose reliability is jointly affected by fluence attenuation, spectral mixing, MPE-limited illumination, and ocular motion—issues that motivate the standardization and validation strategies discussed in later sections.

### 3.1 Deep microvascular visualization beyond the optical scattering limit

PAI commonly uses near-infrared (NIR) excitation (e.g., 680–970 nm) and can achieve imaging depths in the millimeter range; under certain experimental conditions in general tissue settings, penetration may extend toward the centimeter scale. In ophthalmic applications, however, eye-specific laser safety limits and strong ocular and choroidal absorption are expected to impose stricter constraints on deliverable fluence and practical imaging depth. Even within these constraints, PAI may provide greater depth of reach than OCTA for assessing vascular features in thicker choroidal melanomas (e.g., lesions >2 mm), potentially supporting three-dimensional depiction of deep abnormal vascular networks and tumor parenchyma. This depth advantage derives from strong optical absorption contrast with endogenous chromophores, coupled with ultrasound-based detection, which generally experiences lower attenuation than light in tissue (26). Crucially, depth performance claims derived from non-ocular tissues must be interpreted conservatively; ocular maximum permissible exposure limits and pigment-driven fluence loss significantly narrow this theoretical depth advantage *in vivo*.

By depicting spatial heterogeneity of tumor angiogenesis, PAI may add morphological information relevant to intraocular tumor assessment; however, its incremental value for malignancy grading or metastatic risk stratification remains to be established in ophthalmic cohorts. Given that *in vivo* clinical evidence in intraocular tumors remains limited, methodological feasibility is currently supported largely by cross-disciplinary photoacoustic tomography (PAT) studies, including characterization of vascular architecture in deep tumors such as breast and prostate cancer (27) and high-resolution mapping of deep cerebral hemodynamics in small-animal models (28). Direct ophthalmology-specific validation under ocular safety and motion constraints remains necessary (29). Furthermore, increased depth of reach does not automatically translate into clinically actionable information unless image quality, reproducibility, and interpretability remain stable across diverse patient profiles and tumor pigmentation levels.

### 3.2 Oxygen saturation imaging: noninvasive mapping of hypoxia-related features

A key functional capability of multispectral PAI is its ability to exploit wavelength-dependent absorption differences between oxyhemoglobin (HbO_2_) and deoxyhemoglobin (HbR) to estimate microvascular sO_2_. This oxygenation-related information is of particular interest in choroidal melanoma because hypoxia-associated microenvironments have been linked to radioresistance, immune evasion, and metastatic potential (21, 30). The feasibility of PAI for evaluating oxygenation has been demonstrated across ischemic diseases and solid-tumor models. For example, PAI has been used to track hemodynamic and oxygenation changes in experimental ischemic stroke (31) and to detect oxygenation-related microvascular dysfunction in clinical studies of diabetic foot disease (32). In oncology, multispectral PAI incorporating sO_2_-related metrics improved discrimination between benign and malignant ovarian masses (reported area under the receiver operating characteristic curve (AUC) of 0.93) (33) and has been used to longitudinally monitor tumor oxygenation and assess early response to anti-angiogenic therapy in breast cancer models (30). It must be emphasized that “sO_2_” in PAI represents an apparent estimate rather than a direct measurement; its physiological interpretation becomes highly ambiguous when optical fluence varies unpredictably across depth and complex tissue compositions.

Nevertheless, in pigment-rich ocular tissues, quantitative sO_2_ estimation may be particularly sensitive to optical fluence attenuation, spectral interference, and reconstruction bias, and its clinical utility in intraocular tumors requires dedicated validation under ophthalmic imaging conditions (34). Moreover, even if group-level differences are detectable in research settings, the threshold for clinical decision-making in individual patients (e.g., predicting radioresistance) will require clearly defined repeatability criteria and clinically meaningful effect sizes.

### 3.3 Endogenous contrast and pediatric safety considerations

PAI can generate contrast from endogenous chromophores (e.g., hemoglobin in microvessels and melanin in choroidal tissue), enabling high-contrast imaging without ionizing radiation and, in many use scenarios, without exogenous contrast agents. This feature may be advantageous for longitudinal management of pediatric patients with RB. Compared with OCTA, which often requires stable fixation and patient cooperation, and CEUS or MRI, which may require contrast administration (with potential hypersensitivity reactions or other safety considerations such as gadolinium retention), endogenous-contrast PAI may reduce procedural burden during repeated follow-up. However, a “contrast-agent–free” modality does not inherently guarantee a “low-burden” procedure in pediatric oncology; stringent requirements for motion control, patient cooperation, and extended workflow times remain substantial limiting factors for real-world adoption.

Evidence supporting label-free PAI in vulnerable populations has been reported in non-ocular models, including characterization of hematoma morphology and dynamics in a neonatal mouse intracerebral hemorrhage model (35) and evaluation of developmental vascular changes (including sO_2_-related metrics) in fetal studies of alcohol exposure (36). With regard to ocular safety, a preclinical rabbit-eye study reported no detectable retinal or choroidal thermal injury under specific illumination conditions at a laser exposure level of 20 mJ/cm² (20). Such data are informative for system development but do not substitute for human safety validation under clinically relevant parameters and standards. A cautious interpretation is warranted because tumor pigmentation, repeated exposures, and device-specific beam geometry may materially change the safety margin, particularly in melanin-rich lesions.

### 3.4 Dynamic monitoring: toward early assessment of treatment response

**Figure 3:**
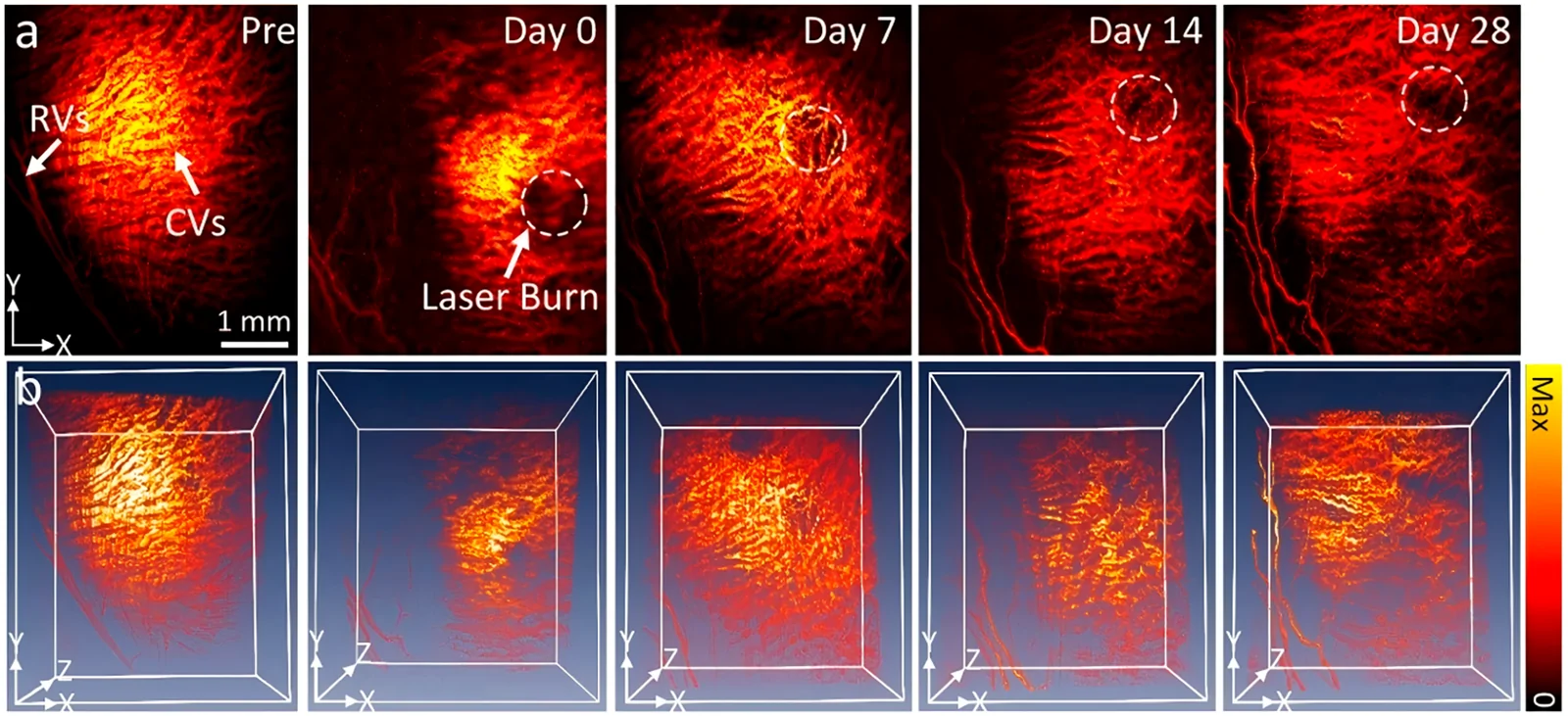
*In vivo* longitudinal photoacoustic monitoring of choroidal and retinal microvascular dynamics (38). **(a)** Serial two-dimensional photoacoustic microscopy (PAM) projection images acquired over a 28-day period from a rabbit model. The sequence tracks morphological and functional changes in retinal vessels (RVs) and choroidal vessels (CVs) prior to (Pre) and following a targeted localized laser burn. The progressive reduction and subsequent remodeling of the vascular signal within the lesion (dashed circles) are clearly visible. **(b)** Corresponding three-dimensional (3D) volumetric PAM reconstructions over the same temporal window. These depth-resolved renderings demonstrate the unique capability of PAI to noninvasively monitor deep-tissue vascular occlusion and healing dynamics over time. Importantly, the “laser burn” denotes the photocoagulation injury used to create the disease model, rather than any iatrogenic tissue damage induced by the PAI illumination itself. This robust longitudinal tracking highlights the potential of PAI for evaluating therapeutic responses in ocular lesions. (Reprinted from (38), under CC BY 4.0.). CVs, choroidal vessels; PAI, photoacoustic imaging; PAM, photoacoustic microscopy; RVs, retinal vessels.

Benefiting from relatively high temporal resolution (e.g., ~10–50 Hz in many conventional systems), PAI can support dynamic and longitudinal tracking of transient hemodynamic and oxygenation-related fluctuations on sub-second timescales (26). Real-time or near-real-time capability has been demonstrated in studies capturing cerebrovascular reactivity and sO_2_ fluctuations following acute alcohol exposure (37), as well as in investigations using exogenous tracers to monitor early microvascular perfusion redistribution during cerebral ischemia (31). A key limitation is that temporal performance reported in non-ocular settings may not translate directly to the eye, where ocular motion, fixation instability, and safety-limited illumination can constrain usable frame rates and effective signal-to-noise ratio (SNR). Despite these technical constraints, PAI has demonstrated robust capabilities for long-term, noninvasive tracking of deep-tissue vascular remodeling and occlusion dynamics over a 28-day period following localized ocular injury, without relying on exogenous contrast agents (**Figure 3**).

In oncology, PAI has been used to monitor perfusion- and oxygenation-related readouts in target lesions, providing functional information that may change before gross morphological shrinkage. For example, in a breast cancer model treated with the anti-angiogenic agent bevacizumab, early changes in hemoglobin-related parameters and oxygenation dynamics enabled differentiation between treatment-responsive and non-responsive phenotypes over weeks of follow-up (30). In intraocular tumors, similar monitoring could help quantify treatment-associated changes in tumor vascularity and hypoxia-related features after local radiotherapy or anti-VEGF therapy, but clinical utility will depend on whether the observed changes are sufficiently repeatable at the individual level to support time-sensitive decisions. Without predefined response thresholds and standardized acquisition protocols, longitudinal trends may be difficult to interpret across devices and sites.

### 3.5 Signal attenuation and SNR optimization under strong optical absorption

Although PAI may extend vascular visualization beyond the penetration limits of OCTA, performance in choroidal melanoma can be challenged by strong optical absorption from high concentrations of melanin and hemoglobin. As light propagates deeper, optical fluence attenuation can weaken photoacoustic signals and reduce SNR, limiting detectable depth and increasing uncertainty in sO_2_ estimation. Importantly, strong superficial absorption can also accentuate depth-dependent bias, creating an apparent “contrast-rich” image that does not necessarily reflect reliable deep-tissue information.

To address these constraints, current development efforts pursue both hardware- and algorithm-oriented strategies. On the hardware side, optimization of excitation wavelength (e.g., shifting toward longer wavelengths, such as 1064 nm where appropriate), system geometry, and the use of photoacoustic computed tomography (PACT) configurations may improve deep-tissue signal recovery (26). Stacked dual-element ultrasound transducers have also been explored to better balance sensitivity and bandwidth for multiscale vascular analysis and oxygenation-related measurements (39). On the algorithm side, spectral- and depth-dependent compensation models have been developed to mitigate fluence-related bias (e.g., approaches reported for ovarian lesions) (40), and deep learning–based reconstruction methods have been investigated to improve image fidelity from noisy or sparsely sampled multispectral data (41). However, improvements reported in controlled settings may degrade in pigment-rich ocular tumors unless the models are validated against realistic ocular geometries, motion conditions, and safety-limited illumination, and unless failure modes are explicitly characterized.

### 3.6 Standardization and gold-standard validation of sO_2_ quantification

A central translational challenge for microenvironmental assessment with PAI is the standardization of sO_2_ quantification. Multispectral sO_2_ estimation is an inverse problem sensitive to unknown and spatially heterogeneous optical fluence; consequently, conventional linear spectral unmixing can be fragile under noise and tissue-dependent scattering and attenuation (40). Different analytical frameworks (e.g., linear fitting and machine learning models) may therefore yield inconsistent results across platforms, which directly limits multicenter comparability. In practice, sO_2_ values that appear numerically precise can still reflect model-dependent bias rather than true physiological differences.

Beyond these general limitations, phantom and simulation studies have shown that strong background absorbers and depth-dependent spectral coloring can introduce substantial bias (potentially on the order of tens of percentage points) in apparent sO_2_ estimation when fluence and spectral interference are not adequately modeled and compensated (42). In melanin-rich ocular tissues, melanin’s broadband absorption can further confound hemoglobin unmixing and may lead to an underestimation of oxygenation in melanotic lesions (25).

To benchmark algorithm responsiveness under controlled physiological perturbations, reversible ischemia paradigms are commonly used—for example, brief forearm cuff occlusion in skeletal muscle (43). Methodological advances have improved robustness; for instance, a convex-cone–based model reported a mean sO_2_ error of ~3% (44), and deep learning frameworks have been explored to suppress reconstruction artifacts in spectroscopic PAT (41). Nevertheless, for intraocular tumors—particularly under strong pigment-related absorption and spectral mixing—there remains a lack of ophthalmology-specific calibration studies benchmarked against credible reference standards. Because direct “gold-standard” oxygen measurements are difficult to obtain *in vivo* in the eye, validation may need to rely on a layered strategy combining tissue-mimicking phantoms with controlled melanin gradients, repeatability testing (test–retest), physiological challenges (e.g., oxygen-breathing paradigms), and opportunistic correlation with histopathologic hypoxia markers when available (13, 20, 21, 45). Without such standardized validation workflows, sO_2_-based endpoints are unlikely to be accepted for routine clinical decision-making.

## 4 Prospects of PAI for high-resolution choroidal microvascular imaging

**Figure 4:**
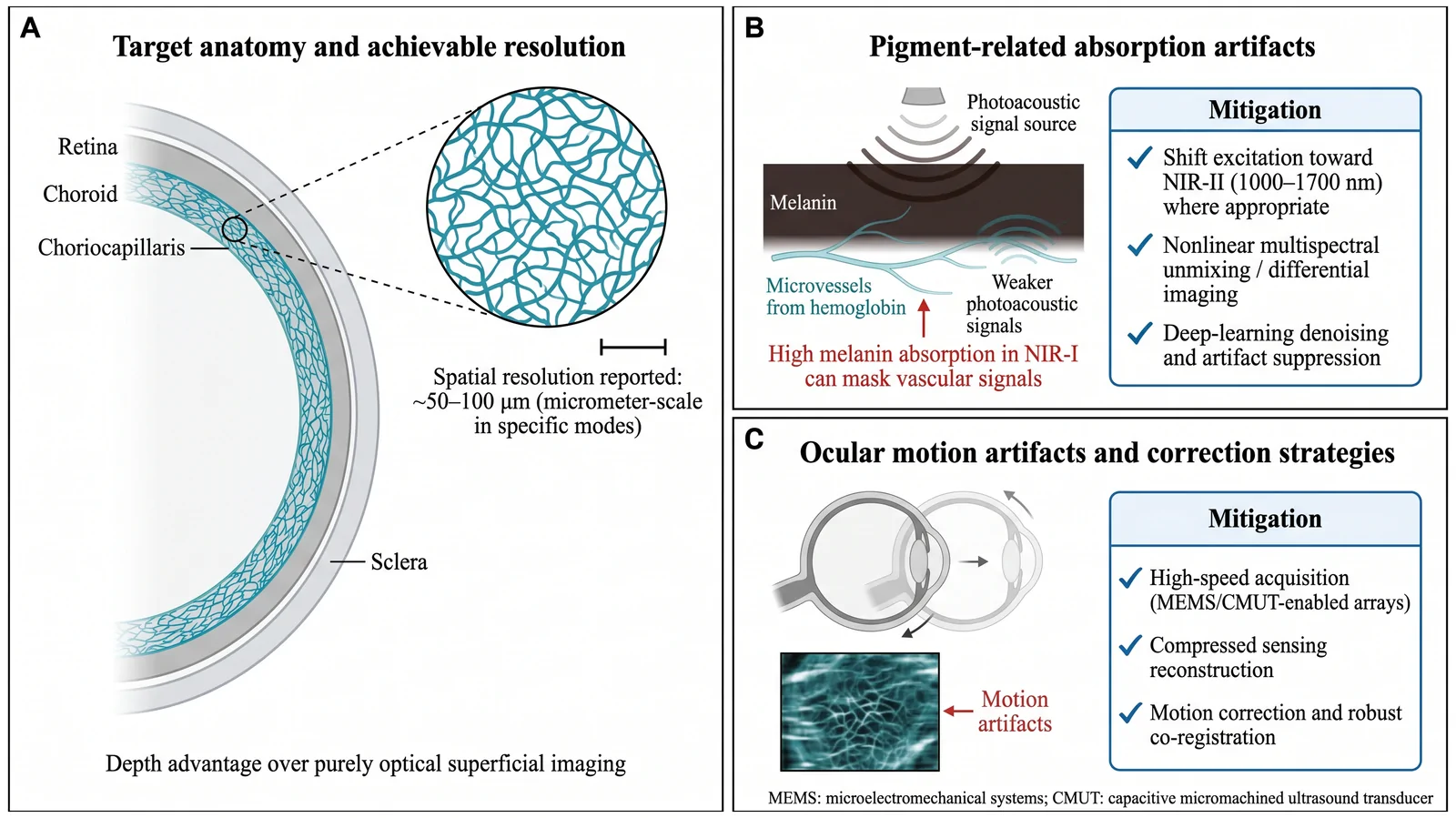
High-resolution choroidal PAI: target anatomy, pigment-related artifacts, motion artifacts, and mitigation strategies. **(A)** Target anatomy for choroidal/choriocapillaris imaging and representative spatial resolution reported in the literature (typically ~50–100 μm; micrometer-scale in selected configurations). **(B)** Pigment-related absorption artifacts: strong melanin absorption in the NIR-I window can dominate background signals and mask hemoglobin-derived microvascular contrast; potential mitigation includes shifting excitation toward NIR-II where appropriate, nonlinear multispectral unmixing/differential imaging, and deep-learning–based denoising/artifact suppression. **(C)** Ocular motion artifacts (e.g., saccades) may cause misregistration and reconstruction errors; mitigation strategies include high-speed acquisition (e.g., MEMS/CMUT-enabled arrays), compressed sensing reconstruction, and eye-tracking–assisted motion correction and robust co-registration. (Schematic generated with the assistance of Gemini 3.1.) NIR-I, first near-infrared window; NIR-II, second near-infrared window; MEMS, microelectromechanical systems; CMUT, capacitive micromachined ultrasound transducer; PAI, photoacoustic imaging.

High-resolution choroidal microvascular imaging with PAI is driven by the need to visualize fine vascular features at depth while maintaining clinically interpretable image fidelity. Key practical challenges include pigment-dominant absorption that can obscure hemoglobin-derived signals and motion-induced artifacts that degrade reconstruction and quantification. These issues and representative mitigation strategies are summarized in **Figure 4**, and they motivate the engineering trade-offs discussed in this section among resolution, depth, and repeatability.

### 4.1 Spatial resolution at depth: toward imaging of the choriocapillaris

**Figure 5:**
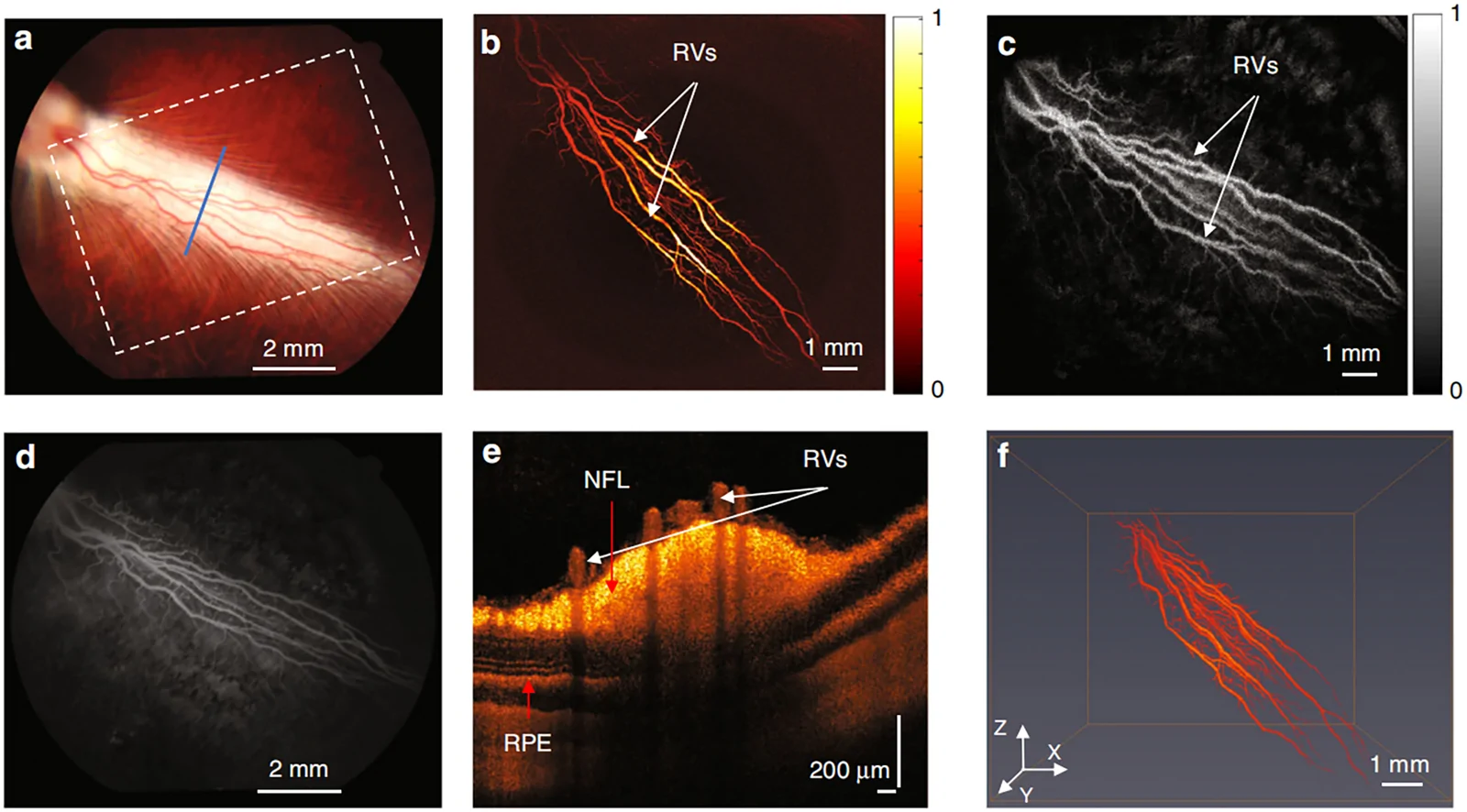
High-resolution *in vivo* multimodal photoacoustic and optical coherence tomography imaging in a pigmented ocular model (46). **(a)** Color fundus photograph of a pigmented rabbit eye. The white dashed box indicates the field of view used for photoacoustic scanning, and the blue line indicates the location of the OCT B-scan. **(b)** Two-dimensional (2D) photoacoustic microscopy (PAM) image acquired within the dashed box in **(a)**, showing retinal vasculature based on endogenous hemoglobin absorption. **(c)** Fluorescence microscopy (FM) image of the same region. **(d)** Corresponding fundus fluorescein angiography (FA) image. **(e)** Co-registered optical coherence tomography (OCT) B-scan acquired along the blue line in **(a)**, showing retinal depth-resolved structural layers; the retinal pigment epithelium (RPE) and nerve fiber layer (NFL) are indicated, and white arrows mark cross-sections of retinal vessels. **(f)** Three-dimensional (3D) reconstruction of PAM data, showing the spatial distribution of retinal vessels. (Reprinted from (46), under CC BY 4.0.) PAM, photoacoustic microscopy; OCT, optical coherence tomography; FM, fluorescence microscopy; FA, fluorescein angiography; RVs, retinal vessels; NFL, nerve fiber layer; RPE, retinal pigment epithelium.

By leveraging the photoacoustic effect, PAI has been explored as a means of improving spatial resolution in probing deep ocular microcirculation (24). In soft tissue, ultrasound propagation generally experiences less scattering than light, which can partially mitigate the signal attenuation encountered by purely optical techniques (e.g., optical coherence tomography (OCT)) in the diffusive regime while preserving biochemical sensitivity via optical absorption contrast (11). In studies of the normal and diseased choroid, PAI has been reported to achieve spatial resolution on the order of ~50–100 μm and, in specific configurations, even at the micrometer scale, thereby enabling visualization of fine vascular architecture, including features attributed to the choriocapillaris (1, 24). A representative *in vivo* example of high-contrast ocular multimodal imaging in a pigmented rabbit eye underscores this capability, successfully resolving complex microvascular networks at depth and demonstrating the complementary structural and absorption-based contrast of OCT and PAI (**Figure 5**). However, reported resolution is highly dependent on imaging mode and experimental conditions, and “choriocapillaris-resolved” claims may be sensitive to interpretation, particularly when validation against an anatomical ground truth is limited.

This level of microvascular detail is typically challenging for conventional ophthalmic ultrasound (limited by acoustic frequency and bandwidth) and for MRI (limited by voxel size). For intraocular solid tumors such as melanoma, OCT retains clear clinical utility for evaluating superficial retinal structures and may provide morphologic features relevant to organ-preserving treatment planning (47). Meanwhile, advances in super-resolution PAI have been investigated to alleviate the classical trade-off between penetration depth and spatial resolution (4). Nonetheless, practical *in vivo* performance in the eye remains constrained by ocular safety limits, pigmentation-related attenuation, and motion, and improvements in nominal resolution may not translate into stable, reproducible readouts across patients and devices.

### 4.2 Integrated structural–functional imaging: coupling vascular morphology with oxygenation-related readouts

A key promise of PAI is the ability to acquire vascular morphology and oxygenation-related functional information within a single noninvasive imaging framework. In choroidal disease, endogenous chromophores—particularly hemoglobin in different oxygenation states—provide strong intrinsic contrast for PAI (24). Analogous to “structure + function” paradigms in other multimodal imaging fields, PAI may help derive a more microenvironment-oriented physiological profile for small intraocular tumors. However, simultaneous acquisition does not guarantee that structural and functional outputs have matched reliability, because functional readouts can be disproportionately affected by fluence heterogeneity and spectral interference in pigment-rich ocular tissues.

In microcirculation studies, PAI has been used to estimate parameters such as total hemoglobin–related signals and the spatial distributions of sO_2_, and to derive additional flow-related indices under specific assumptions and system configurations (1). For fundus masses that may mimic malignant lesions, such as choroidal hemangioma, combining morphological vascular information with oxygenation-related readouts may provide complementary cues for differential diagnosis (48). Importantly, some commonly reported hemodynamic proxies remain model-dependent rather than directly quantitative, and without standardized calibration and repeatability metrics, the risk of over-interpreting small between-group differences remains nontrivial. Head-to-head clinical validation is therefore necessary to establish whether combined readouts improve diagnostic specificity and decision-making beyond conventional imaging.

### 4.3 Differential diagnosis of complex choroidal lesions: potential added value and biological context

In clinical practice, differentiating choroidal vascular lesions can be challenging. For example, atypical choroidal melanoma may be confused with isolated choroidal hemangioma (including lesions associated with Sturge–Weber syndrome) (49) or with rare entities such as ciliary body adenoma of the retinal pigment epithelium (50). Diagnostic challenges have also been reported in atypical presentations (51). In this setting, PAI may offer an additional dimension to lesion characterization by combining vascular architecture information with oxygenation-related readouts. However, vascularity and oxygenation patterns can be heterogeneous and partially overlapping across lesion types, and imaging findings may be confounded by secondary changes (e.g., fibrosis, thrombosis, or inflammation), which can limit specificity if interpreted in isolation.

Angiogenesis in ocular pathology is regulated by molecular pathways. For instance, in choroidal neovascularization, hypoxia-inducible factor-2α *(HIF-2α)* and its downstream target, plasminogen activator inhibitor-1 (PAI-1), have been implicated as molecular drivers (52). Although imaging-based PAI cannot directly measure these targets, mapping hypoxia-related features (e.g., regions with low sO_2_ estimates) may provide indirect, hypothesis-generating information about hypoxia-associated angiogenic activity. Such inference remains indirect and should not be treated as a surrogate for pathway-level measurement. With multi-wavelength excitation and endogenous contrast, functional scanning of lesion margins and cores may help describe microenvironmental heterogeneity and support future studies assessing responses to anti-angiogenic or metabolism-targeted therapies (24).

### 4.4 Pigment-related artifacts and engineering strategies for compensation

Although PAI provides high optical absorption contrast, the anatomy and optical properties of intraocular tumors introduce substantial challenges for reliable signal extraction. Melanin, abundant in choroidal tissue and melanomas, exhibits high optical absorption in the first NIR window (NIR-I) (25). This strong endogenous background absorption can generate large photoacoustic signals that mask weaker hemoglobin-derived signals from microvessels within the same spectral range, producing image artifacts and reducing interpretability of vascular features (25). Importantly, pigment-related interference affects not only vessel conspicuity but also the stability of spectroscopic quantification, because melanin-driven fluence attenuation and spectral overlap can bias hemoglobin-based estimates even when vessels remain visually detectable.

To mitigate pigment-related spectral interference, several engineering strategies are being explored. On the hardware side, shifting excitation toward the second NIR window (NIR-II; 1000–1700 nm) has been proposed. NIR-II excitation has been associated with lower background and improved depth performance in certain tissue settings; however, any potential increase in MPE must be evaluated against ocular safety standards rather than extrapolated from non-ocular applications (53). On the software side, approaches include multispectral nonlinear unmixing and differential imaging to better separate blood-related signals from pigment-dominated backgrounds. Deep learning frameworks have also been investigated for denoising, artifact suppression, and high-fidelity reconstruction in photoacoustic imaging (54). Nevertheless, improved visual quality does not necessarily imply improved quantitative accuracy, and algorithmic gains can be fragile when acquisition conditions, pigmentation, and system geometry differ from the training or calibration settings. A combined “NIR-II–enabled hardware plus advanced computational reconstruction” strategy may help attenuate pigment-related interference in ophthalmic PAI systems, but practical performance and safety boundaries require further validation.

### 4.5 Ocular motion artifacts: hardware–software co-optimization for robust imaging

High-resolution images are important for intraocular tumor assessment; however, unavoidable ocular movements (including saccades) pose a major barrier to clinical translation of PAI. Motion can cause spatial misregistration during acquisition and lead to incomplete datasets, generating reconstruction artifacts under assumptions that require temporal stability (34). Motion is also a potential source of bias for multispectral imaging because wavelength-to-wavelength misalignment can propagate into spectroscopic unmixing errors even when single-wavelength images appear acceptable.

To address motion, current development efforts combine faster acquisition hardware with computational reconstruction. On the hardware side, miniaturized ultrasound transducers enabled by microelectromechanical systems (MEMS) have been explored to facilitate probe miniaturization and endoscopic implementations (55). Multi-view acquisition using flexible capacitive micromachined ultrasound transducer (CMUT) arrays has been proposed to improve field of view, spatial resolution, and contrast, potentially reducing motion sensitivity by shortening acquisition windows (56). On the reconstruction side, compressed sensing can recover higher-quality images from fewer measurements, thereby reducing acquisition time and mitigating motion-related degradation (57). Deep learning models have also been investigated for restoring motion-degraded images and suppressing artifacts (58). However, motion correction methods that work in controlled settings may be less robust in real-world ophthalmic workflows unless they are integrated with reliable eye tracking, standardized protocols, and explicit failure detection. Hardware–software co-optimization is therefore central to improving motion robustness and enabling clinically interpretable choroidal vascular imaging with ophthalmic PAI.

## 5 Multimodal strategies: toward a “vascular–metabolic/oxygenation” diagnostic framework

**Figure 6:**
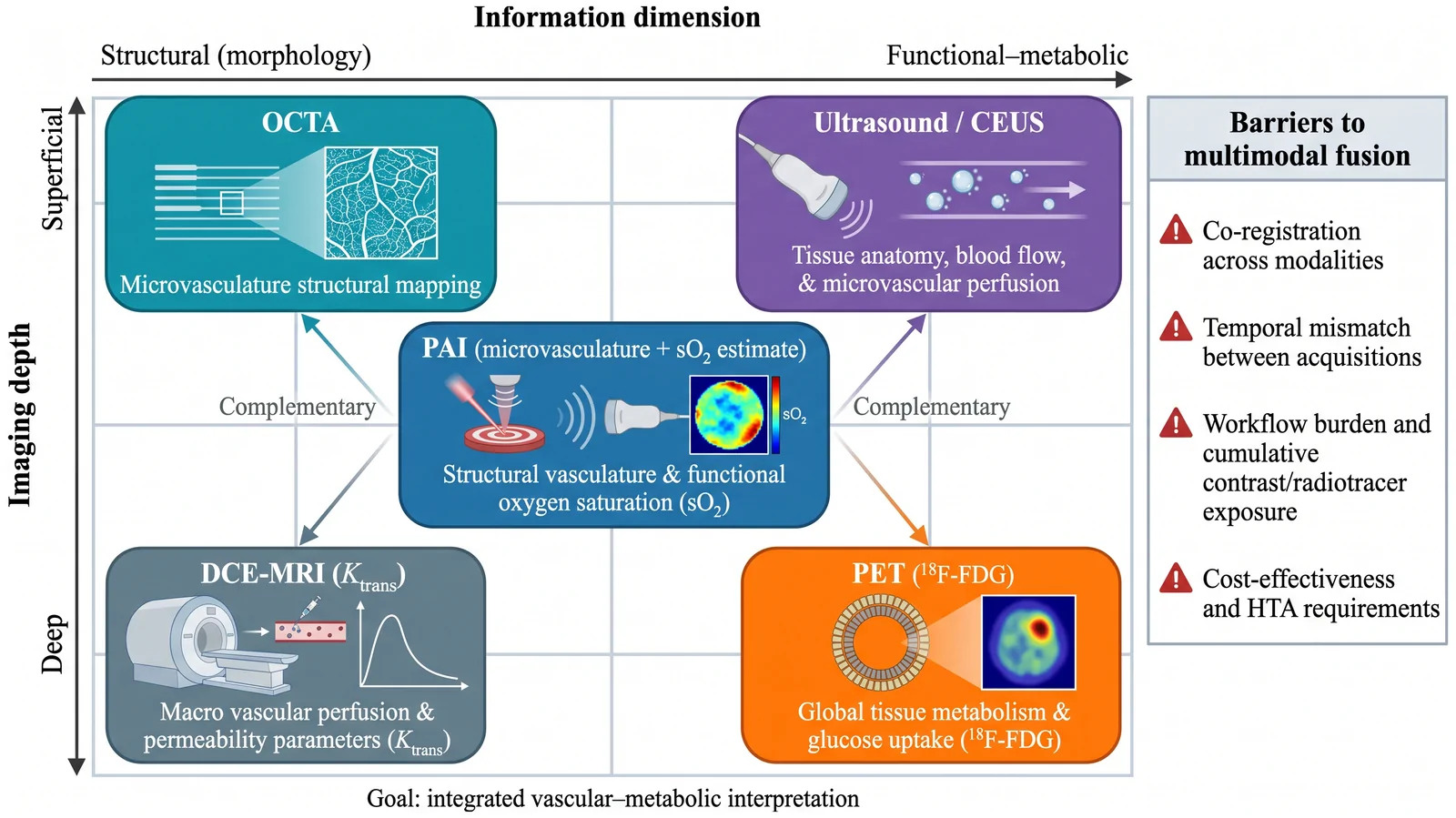
Multimodal “vascular–metabolic/oxygenation” framework: depth-by-information matrix and barriers to fusion. Conceptual positioning of OCTA, ultrasound/CEUS, DCE-MRI, PET, and PAI in a two-dimensional matrix defined by imaging depth (superficial → deep) and information dimension (structural → functional/metabolic). OCTA primarily provides high-resolution superficial microvascular morphology/flow-related contrast; ultrasound/CEUS provides tissue anatomy and predominantly macro-scale perfusion kinetics; DCE-MRI provides macro-perfusion/permeability surrogates (e.g., *K*_trans_); PET reflects metabolic activity such as glucose uptake (e.g., ^18^F-FDG). PAI may bridge these domains by providing absorption-based microvascular contrast and apparent sO_2_ estimation within a depth range constrained by ocular safety and tissue optics. Key barriers to multimodal fusion are summarized, including cross-modality co-registration, temporal mismatch between acquisitions, workflow burden and cumulative contrast/radiotracer exposure, and cost-effectiveness/HTA requirements. (Schematic generated with the assistance of Gemini 3.1.). OCTA, optical coherence tomography angiography; CEUS, contrast-enhanced ultrasound; DCE-MRI, dynamic contrast-enhanced magnetic resonance imaging; PET, positron emission tomography; ^18^F-FDG, ^18^F-fluorodeoxyglucose; PAI, photoacoustic imaging; sO_2_, oxygen saturation (apparent estimate); *K*_trans_, volume transfer constant; HTA, health technology assessment.

No single imaging modality simultaneously provides sufficient depth coverage, microvascular detail, and oxygenation/metabolic information for intraocular tumors. Accordingly, a multimodal framework can be considered along two axes—imaging depth and information dimension (structure → function/metabolism). As summarized in **Figure 6**, major modalities can be positioned within this matrix, highlighting the potential bridging role of PAI for microvasculature and apparent sO_2_ estimation, while underscoring practical fusion barriers including co-registration challenges, temporal mismatch, workflow burden, and HTA requirements.

### 5.1 PAI + OCTA: complementary information from superficial structure to deeper oxygenation-related features

OCTA has established clinical value in ophthalmology and can visualize superficial retinal and choroidal capillary networks at micrometer-scale resolution; however, strong photon scattering in dense tumor tissue limits its ability to interrogate deeper regions. In contrast, PAI leverages ultrasound detection and can provide absorption-based contrast from chromophores such as hemoglobin, with the potential to generate oxygenation-related functional metrics (e.g., sO_2_ estimates) using multispectral acquisition (4, 19). Accordingly, combining OCTA and PAI may provide a more comprehensive assessment that spans superficial vascular morphology, absorption-based vascular contrast, and oxygenation-related information. A practical caveat is that two-modality workflows increase the complexity of acquisition and interpretation, and the incremental benefit must justify the additional equipment, time, and potential motion-related failure modes in routine clinical practice.

From a tumor biology perspective, severe hypoxia can activate hypoxia-inducible pathways (e.g., *HIF-1α* signaling), which are implicated in pathological angiogenesis and adaptive tumor behavior (21). A joint OCTA–PAI approach may therefore be useful for investigating potential spatial relationships between superficial vascular alterations and deeper hypoxia-related features, although such “coupling” patterns and their prognostic value require dedicated clinical validation. Technically, multimodal integration also faces practical barriers, including robust co-registration and field-of-view alignment across systems (59). Even with successful registration, discordant contrast mechanisms may yield seemingly inconsistent findings (e.g., flow-based OCTA dropout versus absorption-based PAI signal), which can complicate interpretation unless prespecified decision rules are developed. Nonetheless, the complementary depth coverage and information content of OCTA and PAI suggest that they may be a promising combined strategy for characterizing the “vascular–hypoxia” landscape in intraocular tumors (11, 60).

### 5.2 PAI + DCE-MRI/ultrasound: integrating macro-perfusion with micro-scale oxygenation heterogeneity

DCE-MRI can provide macro-scale perfusion and permeability information at the whole-tumor level, and parameters such as the volume transfer constant *(K*_trans_) are commonly used as surrogate markers of tissue perfusion and vascular permeability. However, due to limited spatial resolution, MRI may obscure intratumoral micro-scale heterogeneity. Conceptually, combining macro-scale perfusion parameters from DCE-MRI with oxygenation-related maps from PAI could support a “macro–micro” model of tumor perfusion and microenvironmental status. For example, DCE-MRI may suggest globally increased perfusion in choroidal melanoma, whereas the spectroscopic capability of PAI may further indicate that such regions are characterized by structurally abnormal vasculature with heterogeneous oxygenation and focal hypoxia-related features (5, 7). This potential mismatch between perfusion and oxygenation may be relevant to treatment response, although its predictive value requires validation in ophthalmic cohorts. Importantly, adding modalities can also add confounding: differences in patient positioning, acquisition timing, and contrast-agent effects may introduce variability that can mimic biological change if not carefully controlled.

Ultrasound imaging remains widely used in ophthalmology because of its real-time capability. PAI is inherently compatible with ultrasound-based detection and has been considered an emerging ultrasound-related modality for ocular vascular assessment (61). In principle, integrating grayscale ultrasound morphology and PAI oxygenation-related readouts within a single acoustic platform could enable co-registered structural and functional mapping, analogous to multimodal imaging paradigms in other clinical fields (59). Nonetheless, practical implementation will depend on whether combined systems can maintain stable acoustic coupling and reproducible quantitative outputs under routine clinic constraints, rather than only in controlled laboratory settings.

### 5.3 PAI + PET: linking global glucose metabolism with local vascular/oxygenation features

Positron emission tomography (PET), using radiotracers such as ^18^F-fluorodeoxyglucose (^18^F-FDG), enables noninvasive assessment of abnormal glucose uptake at the whole-body or regional level. However, PET has relatively limited spatial resolution for small intraocular lesions and does not directly provide local vascular functional information (e.g., perfusion and oxygenation status) that may underlie metabolic abnormalities. Integrating PET-derived metabolic activity maps with PAI-derived microvascular and oxygenation-related information could, in principle, support a coupled “metabolism–vasculature/oxygenation” interpretation framework (10). In ophthalmology, this approach may be more realistic as a research workflow or as part of broader staging and systemic assessment, rather than for routine primary lesion characterization, given the workflow burden and radiotracer exposure.

This multimodal concept relates to central mechanisms of tumor energy metabolism, including the Warburg effect (62). For instance, PET may identify subregions of choroidal melanoma with increased glucose uptake, while PAI may suggest that these metabolically active areas are associated with hypoxia-related microenvironments (e.g., low sO_2_ estimates), a pattern that is consistent with hypoxia-associated glycolytic reprogramming. Importantly, such inferences remain hypothesis-generating and require dedicated validation. Looking forward, advances in nanomedicine have enabled the development of dual-modality PET/PAI probes in materials and preclinical research (10), which may facilitate more integrated molecular imaging in the future; nevertheless, their safety, feasibility, and clinical value in intraocular tumors must be established before clinical translation. In addition, complex probes may introduce new regulatory and toxicity considerations that could limit near-term applicability, particularly in pediatric contexts.

### 5.4 Technical barriers to multimodal fusion: co-registration and temporal synchronization

Although “vascular–metabolic” multimodal frameworks are conceptually appealing, translating them into routine ophthalmic practice faces substantial technical and operational barriers. A primary challenge is accurate co-registration across modalities. OCTA, MRI, and PET differ markedly in spatial resolution, field of view, and coordinate systems (e.g., millimeter-scale OCTA fields vs. much larger MRI fields of view), and patient repositioning and ocular motion between examinations further complicate high-fidelity image fusion (59). Even small co-registration errors can be clinically consequential when lesions are small and heterogeneous, and automated registration pipelines may fail silently without robust quality control.

A second critical issue is temporal mismatch. OCTA and PAI can typically be acquired in seconds, whereas DCE-MRI and PET often require minutes to tens of minutes. Such differences in acquisition windows can confound the interpretation of dynamic physiological processes, including changes in perfusion and oxygenation following interventions. As emphasized in clinical translation roadmaps, synchronized acquisition, standardized processing, and clinically meaningful interpretation of large, heterogeneous multimodal datasets remain major informatics bottlenecks (9). In addition, multimodal workflows increase visit duration and may add cumulative exposure to contrast agents and/or radiotracers, thereby increasing patient burden and complicating implementation in routine care. A further concern is that increased data volume can create an “interpretation bottleneck,” in which additional parameters do not improve decision-making unless they are linked to prespecified clinical endpoints.

### 5.5 Health economic considerations: balancing incremental diagnostic value and cost-effectiveness (HTA)

Multimodal imaging has substantial potential to improve diagnostic sensitivity and specificity; however, broad clinical adoption of any new technology requires rigorous health technology assessment (HTA) and cost-effectiveness evaluation. As an emerging photoacoustic modality with the potential to characterize microvascular networks in detail (61), PAI currently entails considerable upfront capital investment and ongoing costs for high-dimensional data acquisition, processing, and interpretation. Nevertheless, evidence from other clinical settings suggests that multimodal strategies may yield long-term economic benefits by reducing downstream costs associated with diagnostic uncertainty. Even so, apparent technical superiority does not guarantee value-based adoption if it does not change management decisions or patient outcomes.

For example, an HTA study in the setting of breast mass evaluation reported that adding optoacoustic/ultrasound (OA/US) to grayscale ultrasound resulted in an incremental cost-effectiveness ratio (ICER) of −31,715.82 USD per quality-adjusted life year (QALY) over a 25-year horizon (63). This negative ICER suggests that, in that setting, the multimodal approach could be economically favorable, largely by reducing false-positive assessments and avoiding unnecessary invasive biopsies. However, because intraocular tumors are much less prevalent than breast tumors, their economic models and clinical pathways differ substantially; therefore, indication-specific cost-effectiveness analyses are required before extrapolating these results to ophthalmology (64). These cross-disciplinary findings nonetheless highlight an important principle for ophthalmic translation: rather than emphasizing technology stacking, development of PAI-enabled workflows should prioritize high-value clinical scenarios—such as noninvasive differentiation of suspected malignant choroidal melanoma or early identification of resistance to anti-VEGF therapy—where incremental diagnostic or monitoring information is most likely to translate into measurable clinical and economic benefit.

### 5.6 Illustrative clinical workflow vignette: integrating PAI into the diagnostic pathway

To illustrate how PAI could be integrated into a clinically plausible diagnostic workflow, consider a patient presenting with an indeterminate, heavily pigmented choroidal mass exceeding 3 mm in thickness, accompanied by subretinal fluid. A plausible stepwise workflow is as follows.

1. Baseline structural assessment. B-scan ultrasound is performed to characterize macroscopic lesion dimensions and internal reflectivity, while OCT and OCTA are used to evaluate adjacent retinal morphology and superficial vascular features. In thick, pigment-dense lesions, OCTA signals may not reliably interrogate the deeper tumor compartment, leaving the vascular information at depth incompletely characterized (65).
2. Optional macro-perfusion assessment with MRI. If the lesion remains indeterminate after ultrasound/OCT/OCTA, or if whole-tumor perfusion/permeability characterization is clinically relevant, DCE-MRI may be performed to provide macro-scale perfusion surrogates (e.g., *K*_trans_), while recognizing its spatial/temporal constraints for small intraocular lesions (22).
3. PAI as a targeted adjunct under ocular safety constraints. When deep vascular information or oxygenation-related heterogeneity is clinically relevant but not accessible with OCTA, PAI may be performed as an adjunct using a safety-compliant illumination protocol and co-registered ultrasound guidance. Under these constraints, PAI may provide additional depth-sensitive absorption contrast and, with multispectral acquisition, generate apparent sO_2_ estimates. Importantly, any low-sO_2_ regions should be interpreted with caution because they may reflect fluence-related bias or pigment-driven spectral interference rather than true hypoxia.
4. Integrated interpretation and triage (hypothesis-generating). A combined OCTA–MRI–ultrasound–PAI dataset may offer complementary cues: OCTA for superficial flow-related features, DCE-MRI for macro-perfusion/permeability surrogates, ultrasound for gross morphology, and PAI for absorption-based vascular contrast and oxygenation-related heterogeneity. Rather than serving as a standalone diagnostic determinant, PAI-derived features could be evaluated as a potential adjunct to raise or lower suspicion for malignancy and to support triage decisions (e.g., intensified follow-up, referral to an ocular oncology center, or proceeding to definitive therapy such as episcleral plaque brachytherapy), pending validation in ophthalmology-specific cohorts.
5. Research endpoints for future validation. In prospective studies, this scenario can be operationalized using prespecified endpoints, such as test–retest repeatability of vascular/sO_2_ metrics, incremental diagnostic accuracy beyond standard imaging, and early post-treatment changes in PAI-derived readouts following radiotherapy or other interventions (66). Any proposed association between oxygenation-related patterns and radiotherapy response should be treated as a research hypothesis and validated against clinically meaningful outcomes.

This scenario highlights a realistic positioning of PAI as a selective, safety-constrained adjunct designed to address deep-tissue information gaps, while emphasizing that clinical value depends on rigorous validation, standardized protocols, and predefined decision thresholds.

## 6 Evidence gaps, validation priorities, and translational pathways for ophthalmic PAI

**Figure 7:**
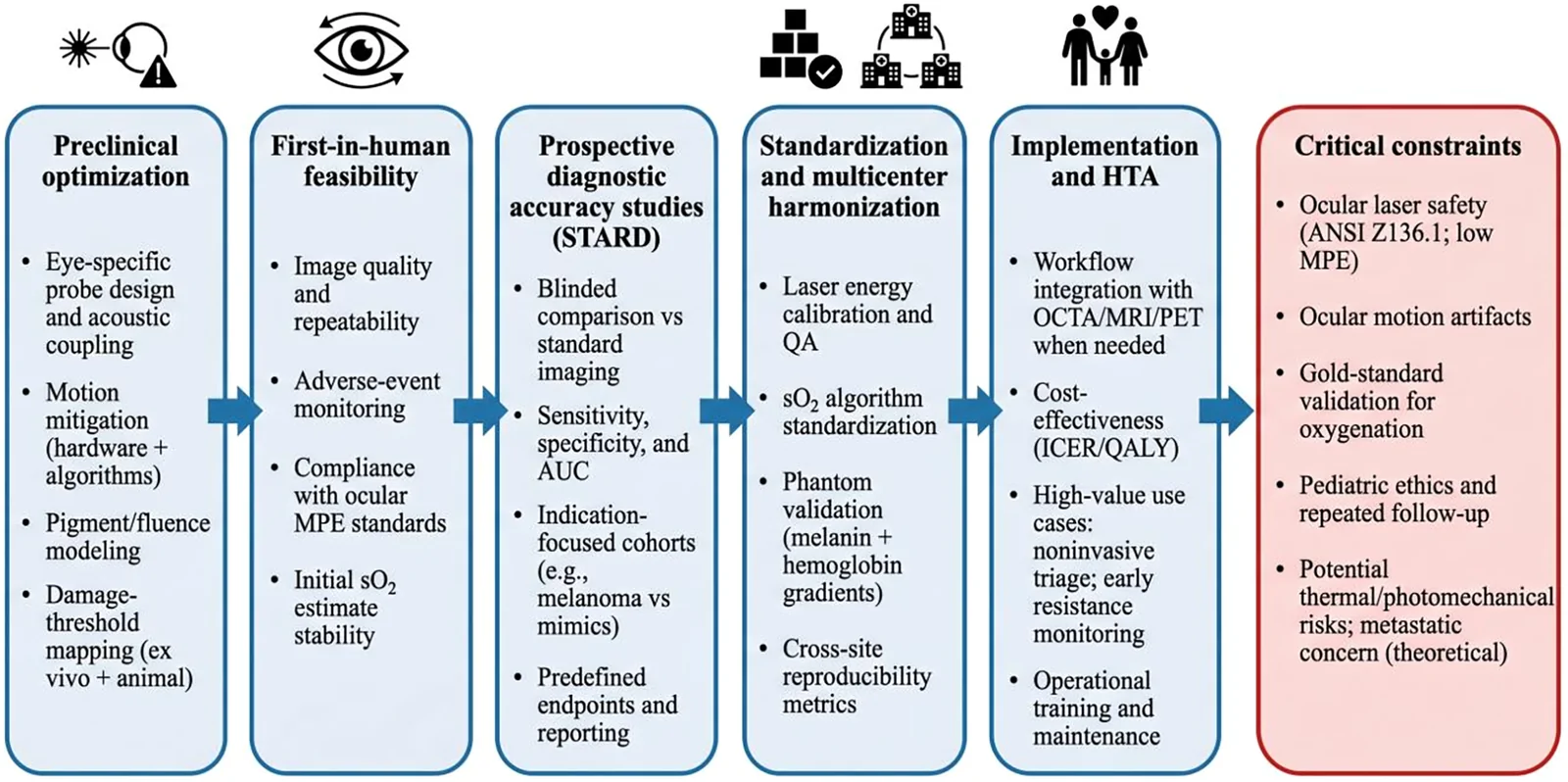
Stage-gated roadmap for clinical translation of ophthalmic PAI: evidence requirements and critical constraints. Stage-gated roadmap for translating ophthalmic PAI into clinical practice. Key phases include: (i) preclinical optimization (eye-specific probe design/acoustic coupling, motion handling, pigment/fluence modeling, and damage-threshold mapping); (ii) first-in-human feasibility (image quality and repeatability, adverse-event monitoring, compliance with ocular MPE standards, and initial stability of apparent sO_2_ estimation); (iii) prospective diagnostic accuracy studies following STARD (blinded comparison vs standard imaging, predefined endpoints, and reporting of sensitivity/specificity/AUC in indication-focused cohorts); (iv) standardization and multicenter harmonization (laser energy calibration and QA, sO_2_ algorithm standardization, phantom validation with melanin/hemoglobin gradients, and cross-site reproducibility metrics); and (v) implementation and HTA (workflow integration, cost-effectiveness evaluation using ICER/QALY, high-value use cases, and operational training/maintenance). Critical constraints are summarized on the right, including stringent ocular laser safety (ANSI Z136.1 and low MPE), ocular motion artifacts, lack of gold-standard oxygenation validation, pediatric ethics and the burden of repeated follow-up, and potential thermal/photomechanical risks. (Schematic generated with the assistance of Gemini 3.1.). PAI, photoacoustic imaging; sO_2_, oxygen saturation (apparent estimate); MPE, maximum permissible exposure; ANSI, American National Standards Institute; STARD, Standards for Reporting Diagnostic Accuracy Studies; AUC, area under the curve; HTA, health technology assessment; ICER, incremental cost-effectiveness ratio; QALY, quality-adjusted life year; QA, quality assurance.

Translating ophthalmic PAI into routine practice requires coordinated progress in safety-compliant illumination, motion-robust acquisition, and standardized oxygenation quantification, followed by staged clinical evidence generation and health-technology assessment. As summarized in **Figure 7**, these requirements define a stage-gated evidence-generation pathway—from preclinical engineering optimization to first-in-human feasibility, prospective diagnostic accuracy studies (STARD), multicenter standardization, and real-world HTA—while highlighting ophthalmology-specific constraints, including stringent MPE-limited illumination, motion artifacts, pigment/fluence-related bias, pediatric ethical considerations, limited gold-standard oxygenation validation, and potential thermal/photomechanical risks.

### 6.1 Engineering translation from animal models to human studies

At present, PAI research in intraocular tumors remains largely at the preclinical stage (e.g., proof-of-concept studies in rabbit models of orthotopic melanoma), and large-scale human clinical data are still lacking. This gap underscores substantial engineering and translational challenges that must be addressed. First, uncontrolled ocular motion (including saccadic movements) is a major acquisition bottleneck because it can cause spatial misregistration and compromise image fidelity and the reliability of sO_2_ estimation. Second, ocular laser safety constraints impose strict physical limits. According to the American National Standards Institute (ANSI) Z136.1 standard, ocular exposure must comply with stringent safety limits; for example, the maximum permissible single-pulse exposure can be as low as 162 nJ for a collimated Gaussian beam under specific conditions (29). This is far below the excitation energies used in many conventional small-animal PAI systems. To maintain an adequate signal-to-noise ratio under low-exposure conditions, exogenous contrast agents with high optical absorption cross-sections (e.g., PAtrace) are being explored as a potential compensatory strategy (67). However, introducing exogenous agents may partially offset the practical advantage of label-free imaging and can add regulatory and workflow complexity, particularly for repeated follow-up or pediatric applications.

In addition, probe miniaturization and conformal design represent another major engineering challenge. Because of the confined orbital space and the curvature of the posterior pole, conventional handheld or linear-array probes may not achieve stable acoustic coupling. In this context, design concepts from raster-scan optoacoustic mesoscopy (RSOM) in dermatology—where miniaturized probes have been developed for clinical translation—may serve as useful engineering reference points (68). Developing dedicated, conformal ophthalmic probes with integrated motion-tracking and compensation algorithms will be essential to advancing PAI from preclinical feasibility to human studies. A practical constraint is that increased miniaturization can reduce acoustic aperture or sensitivity, and engineering gains in one dimension (e.g., footprint) may introduce trade-offs in another (e.g., field of view or quantitative stability).

### 6.2 Systematic bias in reconstruction/unmixing and the lack of multicenter validation

A key perceived advantage of PAI for tumor microenvironment assessment is its ability to quantify oxygenation-related metrics such as sO_2_; however, solving this multispectral inverse problem relies heavily on the robustness of unmixing and reconstruction algorithms. At present, there is no universally accepted standard for acoustic noise suppression, tissue scattering correction, or optical fluence compensation, resulting in substantial variability across algorithms and platforms. For example, conventional linear fitting often assumes spatially homogeneous optical properties—an assumption that is frequently violated in melanin-rich choroidal melanoma—thereby introducing systematic bias. To address these limitations, deep learning–based reconstruction strategies are being actively explored; for instance, conditional generative adversarial networks (cGANs) have been used to perform end-to-end recovery of tissue oxygenation images from multispectral data, thereby improving oxygenation quantification (69). Nevertheless, algorithmic performance can be sensitive to domain shift, and models trained on non-ocular datasets or simplified phantoms may fail unpredictably in pigment-rich, motion-prone ocular settings unless explicitly validated and monitored.

Algorithmic advances alone are insufficient to establish clinical trust. Standardized calibration and validation protocols are essential, including the development of tissue-mimicking phantoms with known hemoglobin oxygenation states and controlled melanin gradients, and rigorous agreement testing between PAI-derived microvascular sO_2_ metrics and credible reference measurements (e.g., invasive tissue oxygen partial pressure microelectrodes). Such steps are necessary to ensure reproducibility in the quantification of intraocular oxygenation and to enable reliable multicenter studies. A further limitation is that overly optimized pipelines may preferentially report “clean-looking” images while obscuring uncertainty; therefore, uncertainty quantification and failure detection should be considered alongside point estimates in translational workflows.

### 6.3 Evidence-based, staged validation pathway for clinical translation

Clinical implementation of PAI in ophthalmology should follow a staged, evidence-based translational pathway to ensure safety and effectiveness. In the first stage, PAI can be embedded as an exploratory research tool within existing clinical trial cohorts. For example, in breast cancer, PAI has been used to capture early radiotherapy-induced remodeling of tumor oxygenation-related microenvironmental features (70). This stage primarily aims to determine whether PAI can provide incremental functional information beyond OCTA or MRI, and to identify which readouts are sufficiently stable for longitudinal tracking. It is also important to recognize that “incremental parameters” do not necessarily translate into better decisions unless their relationship with clinical endpoints is prespecified and reproducible.

In the second stage, rigorously designed prospective diagnostic studies are needed to objectively evaluate the independent clinical value of PAI for differentiating intraocular tumors. For clinically challenging cases that are difficult to characterize using conventional ultrasound alone (e.g., retinoblastoma versus uveal melanoma), studies should be designed and reported in accordance with the Standards for Reporting Diagnostic Accuracy Studies (STARD) guidelines, ideally with blinded assessment (71). Early ophthalmic investigations have reported photoacoustic feature patterns in intraocular tumors, supporting feasibility and highlighting the need for larger, standardized diagnostic cohorts (72). However, diagnostic accuracy estimates can be inflated by spectrum bias and small-sample effects, and multicenter designs with prespecified thresholds are preferable when feasible.

In the third stage, once diagnostic performance and clinical utility are established, real-world health technology assessment (HTA) should be conducted. This stage should evaluate the health economic value of PAI, including whether it reduces unnecessary intraocular biopsy procedures, optimizes follow-up pathways, or improves the cost-effectiveness of expensive targeted therapies. Prioritizing validation in high-impact clinical scenarios (e.g., early confirmation of malignant intraocular tumors) may accelerate regulatory approval and clinical adoption.

### 6.4 Current ophthalmic evidence on intraocular tumors and scope of extrapolation

To contextualize the staged validation pathway, it is necessary to summarize the current ophthalmic intraocular tumor evidence and delineate the scope of extrapolation from the non-ocular optoacoustic literature. Although the biophysical rationale for PAI in pigment-rich, vascular intraocular tumors is compelling, the ophthalmic tumor-specific peer-reviewed evidence remains limited and is largely feasibility-oriented. Based on the ophthalmology-relevant literature identified in this review, the evidence most directly informing intraocular tumor translation can be broadly categorized into the following layers.

- Tumor feasibility evidence. Early ophthalmic investigations have reported photoacoustic feature patterns in intraocular tumors (e.g., retinoblastoma and uveal melanoma), supporting feasibility and motivating larger, standardized diagnostic cohorts rather than the establishment of validated clinical biomarkers (72).
- Ocular safety evidence in tumor-relevant contexts. Preclinical safety evaluation in rabbit eyes reported no detectable retinal or choroidal thermal injury at an exposure level of 20 mJ/cm² under specified illumination conditions, providing early safety information for system development but not substituting for human safety validation (20).
- Enabling evidence under stringent ocular exposure constraints. Ultralow-energy ocular PAM has been reported, supporting the technical feasibility of obtaining ocular photoacoustic signals under strict MPE-limited conditions, but not addressing tumor-specific diagnostic performance (29).
- Enabling evidence for longitudinal ocular vascular monitoring. *In vivo* longitudinal PAM monitoring in a rabbit ocular injury model demonstrated noninvasive tracking of choroidal/retinal microvascular remodeling dynamics over 28 days, illustrating the feasibility of repeated ocular photoacoustic follow-up workflows without exogenous contrast agents, although this model is not an intraocular tumor cohort (38).

Given the limited number of ophthalmic tumor-specific reports currently available, cross-disciplinary optoacoustic studies cited throughout this review are used primarily to support methodological concepts (e.g., multispectral acquisition/unmixing, fluence-compensation strategies, motion-robust reconstruction, and longitudinal monitoring paradigms) rather than to imply equivalent clinical performance in intraocular tumors. Importantly, ocular applications impose distinct constraints—including MPE-limited illumination, pigmentation-related fluence attenuation/spectral mixing, and unavoidable eye motion—that can materially affect signal detectability and the interpretability of apparent sO_2_ estimates. Therefore, extrapolations beyond ophthalmic tumor data should be treated as hypothesis-generating and require ophthalmology-specific validation. Together, these evidence gaps highlight ocular laser safety and dose standardization as key determinants of whether PAI can be translated into routine intraocular tumor workflows.

### 6.5 Ocular laser safety constraints and assessment of iatrogenic thermal/mechanical risks

Retinal laser safety is a major technical and ethical bottleneck for the clinical translation of PAI. Ocular exposure must comply with stringent limits specified by ANSI and related MPE standards, which depend on wavelength, pulse duration, repetition rate, beam geometry, retinal spot size, and exposure duration. These constraints inherently limit deliverable optical energy, creating a trade-off between safe illumination and the signal-to-noise ratio required for deep imaging. Low optical fluence is further attenuated as it traverses pigment-rich choroidal tissue, resulting in weak deep signals and increasing susceptibility to depth-dependent bias. Importantly, “compliance with MPE” should not be interpreted as “uniform safety” across patients, because melanin burden, local focusing conditions, and repeated exposures may materially alter the effective margin of safety in specific lesion phenotypes.

In superficial tissues (e.g., the limb), the feasibility of PAI for tracking oxygenation dynamics has been demonstrated using controlled *in vivo* physiological perturbation paradigms (43). In addition, a preclinical rabbit-eye safety study reported no detectable retinal or choroidal thermal injury under specific illumination conditions at an exposure level of 20 mJ/cm² (20). However, intraocular melanoma contains abundant melanin granules with strong near-infrared photothermal conversion capacity (24). Under certain parameter settings (e.g., higher energy density, specific wavelengths, or repeated exposures), localized heat accumulation and rapid thermoelastic expansion could increase the risk of thermal injury, photomechanical effects, or other tissue responses. Although currently unproven, potential iatrogenic perturbation of fragile neovascular beds in tumors has also been proposed as a concern that warrants experimental evaluation rather than assumption. A practical limitation is that many preclinical safety demonstrations are not directly comparable across systems because spot size, pulse structure, retinal irradiance distribution, and exposure definitions may differ, making “safe” settings difficult to translate without standardized reporting.

A quantitative safety framework can help structure future validation. For example, under simplified assumptions, the upper bound of transient temperature rise can be parameterized as a function of local absorption and delivered fluence (e.g., ΔT scaling with local optical absorption and fluence and inversely with tissue heat capacity), while repeated pulses introduce the possibility of cumulative heating when the pulse repetition interval is shorter than the local thermal relaxation time (73). In parallel, the likelihood of photomechanical effects depends on pulse duration relative to stress confinement and local energy deposition rates, which may be amplified by highly absorbing melanin-rich microdomains (74). Such parameterized models are not substitutes for biological validation, but they provide a falsifiable framework for defining “safe operating windows” and identifying worst-case conditions to test first, rather than relying on qualitative arguments alone.

Accordingly, detailed ex vivo and *in vivo* studies—including large-animal models where feasible—are needed to quantify tissue damage thresholds across pulse widths, repetition rates, beam geometries, and wavelength combinations, and to define an ophthalmology-specific safe operating window that supports functional imaging while minimizing safety risks (9). In addition to histologic injury endpoints, safety assessments should consider repeat-exposure protocols that reflect longitudinal monitoring use cases, because cumulative dose and repeated alignment variability may represent realistic clinical scenarios. To place ocular safety considerations in the broader translational context, **Table 3** summarizes representative evidence and measurable endpoints supporting staged adoption of ophthalmic PAI, spanning diagnostic discrimination, longitudinal monitoring, validation paradigms, motion robustness, and health technology assessment.

**Table 3:** Evidence map supporting translational ophthalmic PAI.

Context	Model/setting	Endpoint(s)	Key findings	Ocular relevance	Ref
Benign vs malignant	Ovarian masses	Apparent sO_2_	AUC improved to 0.93 with sO_2_	Diagnostic value beyond morphology	(33)
Therapy monitoring	Breast cancer (bevacizumab)	Hb & sO_2_ dynamics	Differentiates responders early	Functional change precedes size change	(30)
MV dysfunction	Diabetic foot	O_2_-related MV	Detects hypoxia-associated MV dysfunction	Noninvasive O_2_ impairment detection	(32)
Hemodynamics	Ischemic stroke	Perfusion & sO_2_	Real-time tracking of hemodynamics	High-TR monitoring for treatment assessment	(31)
Longitudinal tracking	Rabbit ocular injury	MV remodeling	28-day tracking of MV remodeling/occlusion	Feasibility for longitudinal ocular workflows	(38)
Preclinical safety	Rabbit eye	Thermal injury	No thermal injury at 20 mJ/cm²	Informs ophthalmic exposure windows	(20)
Low-fluence feasibility	*In vivo* eye	Image quality	Ultralow-energy PAM reported	Feasibility under strict MPE constraints	(29)
Algorithm benchmark	Forearm ischemia	sO_2_ responsiveness	*In vivo* perturbation model	Standardized validation workflow template	(43)
Improved sO_2_ accuracy	Convex-cone model	sO_2_ error	Mean error reduced to ~3%	Reduces bias; motivates standardization	(44)
Phantom validation	Tissue phantoms	Oximetry validation	Absorption/spectral coloring bias sO_2_	Guides calibrated phantoms for ophthalmic sO_2_	(42)
Sparse data recovery	DL (undersampled PAT)	Artifact suppression	DL recovers undersampled data	Relevant for motion-limited/low-fluence scans	(41)
Pigment mitigation	NIR-II window	Background reduction	NIR-II reduces background	Addresses melanin absorption & safety constraints	(53)
Motion robustness	Hardware/algorithm	Artifact mitigation	MEMS/CMUT, CS, & DL improve robustness	Addresses saccade artifacts (key bottleneck)	(55–58)
Artifact taxonomy	Review	Origins & mitigations	Summarizes artifact mitigations	Guides handling of ocular artifacts	(34)
Safety modeling	Retinal dosimetry	Physical endpoints	Heating relates to PRI; photomechanical risk to pulse duration	Parameterized safety framework for melanin	(73, 74)
HTA	Breast mass (OA/US)	Cost-effectiveness	ICER −$31k/QALY (25 years)	Suggests economic value; needs ocular modeling	(63)
Tumor feasibility	RB & uveal melanoma	PAI features	PAI features reported in ocular tumors	Direct tumor feasibility; motivates larger cohorts	(72)

Quantitative values are reported only when explicitly stated in the main text; otherwise, findings are summarized qualitatively to avoid over-extrapolation.

AUC, area under the curve; CMUT, capacitive micromachined ultrasound transducer; CS, compressed sensing; DL, deep learning; Hb, hemoglobin; HTA, health technology assessment; ICER, incremental cost-effectiveness ratio; MEMS, microelectromechanical systems; MPE, maximum permissible exposure; OA/US, optoacoustic/ultrasound; PAM, photoacoustic microscopy; PAT, photoacoustic tomography; PRI, pulse repetition interval; QALY, quality-adjusted life year; RB, retinoblastoma; sO_2_, oxygen saturation; TR, temporal resolution; MV, microvasculature; NIR-II, second near-infrared window; O2, oxygen; PAI, photoacoustic imaging.

### 6.6 Clinical positioning of PAI relative to OCTA

Although PAI offers promising depth penetration and oxygenation-related functional mapping capabilities, it is premature to conclude that PAI will replace OCTA as a new gold standard for choroidal vascular imaging. OCTA has established a strong clinical foundation due to its noninvasiveness, rapid acquisition, high superficial resolution, and extensive evidence base across common retinal vascular disorders. Similar to findings in breast imaging, where multimodal optoacoustic/ultrasound has been positioned as complementary rather than strictly substitutive of standard imaging (13), PAI in ocular imaging is more appropriately viewed as an adjunct in selected scenarios. A further practical consideration is that adding a second modality can increase workflow complexity, and clinical value will depend on whether incremental information changes management decisions rather than simply adding parameters.

For example, in central serous chorioretinopathy (CSC) or in thicker malignant tumors, OCTA may be limited in its ability to characterize deeper choroidal pathology, whereas PAI may provide additional oxygenation-related information to support mechanistic interpretation of disease processes. Future research should therefore focus on head-to-head comparisons to define the respective strengths, failure modes, and optimal indications of each modality. Clarifying where PAI provides unique clinical value—particularly when deeper oxygenation-related information is needed and can be obtained with acceptable repeatability under MPE constraints—will be critical to establishing its role in modern ophthalmic imaging workflows.

## 7 Conclusion

PAI has the potential to complement conventional ophthalmic imaging by extending access to deeper microvascular features and providing oxygenation-related functional readouts (e.g., sO_2_ estimates), thereby addressing aspects of the tumor microenvironment that are difficult to interrogate with existing modalities alone. However, sO_2_ should be interpreted as an apparent estimate that may be biased by fluence heterogeneity, pigment-related spectral interference, and motion, particularly in melanin-rich tumors. Clinical translation will therefore depend on rigorous ophthalmology-specific validation, standardized quantification, and appropriate multimodal integration with OCTA, MRI, and PET, while meeting stringent ocular safety requirements and addressing pediatric ethical considerations. If these barriers are addressed through well-designed prospective studies, PAI may ultimately serve as a valuable complementary tool for diagnosis and longitudinal monitoring in selected intraocular tumor scenarios.
