# Supplementary material for: Potential of photoacoustic imaging for assessing tumor perfusion in intraocular tumors and high-resolution choroidal microvascular imaging: from technical advances to clinical translation
Source: Front Oncol. 2026 Aug 24;16:1901235. doi: 10.3389/fonc.2026.1901235 (PMC13548804; doi:10.3389/fonc.2026.1901235)
Supplement: Supplementary file 1 [file Table1.doc]

Supplementary Table 1. Search strategy for Embase database

| No. | Search terms |
| --- | --- |
| #1 | photoacoustics'/exp |
| #2 | eye tumor'/exp |
| #3 | retinal tumor'/exp |
| #4 | uveal tumor'/exp |
| #5 | photoacoustic imaging':ti,ab OR 'photoacoustic tomography':ti,ab OR 'photoacoustic microscopy':ti,ab OR 'photoacoustic ophthalmoscopy':ti,ab OR 'pa imaging':ti,ab OR 'pai':ti,ab OR 'pat':ti,ab OR 'photoacoustic':ti,ab OR 'optoacoustic imaging':ti,ab OR 'optoacoustic tomography':ti,ab OR 'photo-acoustic':ti,ab OR 'photo acoustic':ti,ab |
| #6 | intraocular tumor*':ti,ab OR 'intra-ocular tumor*':ti,ab OR 'eye tumor*':ti,ab OR 'ocular tumor*':ti,ab OR 'retinoblastoma':ti,ab OR 'uveal melanoma':ti,ab OR 'choroidal melanoma':ti,ab OR 'choroidal hemangioma':ti,ab OR 'choroidal metastasis':ti,ab OR 'intraocular neoplasm*':ti,ab OR 'ocular neoplasm*':ti,ab |
| #7 | #1 and #5 |
| #8 | #2 or #3 or #4 or #6 |
| #9 | #7 and #8 |
