# Supplementary material for: Potential of photoacoustic imaging for assessing tumor perfusion in intraocular tumors and high-resolution choroidal microvascular imaging: from technical advances to clinical translation
Source: Front Oncol. 2026 Aug 24;16:1901235. doi: 10.3389/fonc.2026.1901235 (PMC13548804; doi:10.3389/fonc.2026.1901235)
Supplement: Supplementary file 2 [file Table2.doc]

Supplementary Table 2. Search strategy for Web of Science database

| No. | Search terms |
| --- | --- |
| #1 | TS=("Photoacoustics") |
| #2 | TS=("Eye Neoplasms") |
| #3 | TS=("Retinal Neoplasms") |
| #4 | TS=("Uveal Neoplasms") |
| #5 | TS=("Photoacoustic Imaging" OR "Photoacoustic Tomography" OR "Photoacoustic Microscopy" OR "Photoacoustic Ophthalmoscopy" OR "PA Imaging" OR "PAI" OR "PAT" OR "Photoacoustic" OR "Optoacoustic Imaging" OR "Optoacoustic Tomography" OR "Photo-acoustic" OR "Photo acoustic") |
| #6 | TS=("Intraocular Tumor*" OR "Intra-ocular Tumor*" OR "Eye Tumor*" OR "Ocular Tumor*" OR "Retinoblastoma" OR "Uveal Melanoma" OR "Choroidal Melanoma" OR "Choroidal Hemangioma" OR "Choroidal Metastasis" OR "Intraocular Neoplasm*" OR "Ocular Neoplasm*") |
| #7 | #1 and #5 |
| #8 | #2 or #3 or #4 or #6 |
| #9 | #7 and #8 |
